# Dietary Microplastics Engage Gut Mechanosensory-Endocrine Signaling to Disrupt Bone Homeostasis

**DOI:** 10.64898/2026.04.03.716216

**Published:** 2026-04-07

**Authors:** Aaron S. Romero, Sumira Phatak, Sanchiti Patil, Hamid Y. Dar, Jaclyn A. Rivas, Olufunmilola M Oyebamiji, Brianna B. Maes, Siem S. Goitom, Crystal Madera Enriquez, Jazmin Orozco, Cristina N. Coffman, Ruixuan Liu, Julie G. In, Matthew J. Campen, Kel Cook, Richard Levenson, Jessica M. Gross, Shuguang Leng, Anthony Cretara, Roberto Pacifici, Eliseo F. Castillo

**Affiliations:** 1 Division of Gastroenterology and Hepatology, Department of Internal Medicine, University of New Mexico Health Sciences Center, Albuquerque, New Mexico, USA; 2 Division of Endocrinology, Metabolism and Lipids, Emory University School of Medicine, Atlanta, Georgia, USA; 3 Department of Internal Medicine, University of New Mexico Health Sciences, Albuquerque, NM 87131, USA; 4 Department of Pathology and Laboratory Medicine, University of California Davis Health, Davis, California 95616, USA; 5 Clinical and Translational Science Center, University of New Mexico Health Sciences Center, Albuquerque, New Mexico, USA; 6 Comprehensive Cancer Center, University of New Mexico Health Sciences, Albuquerque, NM 87131, USA; 7 Department of Pathology, University of New Mexico Health Sciences Center, Albuquerque, New Mexico, USA; 8 Authors contributed equally

**Keywords:** Microplastics, serotonin, bone loss, environmental toxicants

## Abstract

Microplastics (MPs) are pervasive environmental contaminants and an emerging component of the human diet^1, 2^, yet their physiological effects remain poorly defined. Here we show that MPs are detectable in mineralized human bone under non-iatrogenic conditions and impair osteoblast mineralization in a donor-dependent manner. Using a physiologically relevant dietary exposure model, we demonstrate that chronic MP ingestion induces sex- and diet-dependent bone loss in mice, predominantly affecting trabecular architecture, in the absence of intestinal pathology or systemic inflammatory cytokine elevation. Instead, MP exposure selectively enhances gut-derived serotonergic signaling, with increased abundance and activity of enteroendocrine cells without evidence of lineage reprogramming. Single-nuclei transcriptomic profiling of the colon resolves enterochromaffin cells and serotonergic target expression across epithelial and enteric neuronal compartments, revealing discrete mechanosensory adaptations without inflammatory activation. Together, these findings implicate ingestion of MPs as bioactive dietary contaminant that disrupt gut-endocrine communication and compromise skeletal homeostasis, uncovering a previously unrecognized pathway linking environmental plastic exposure to bone health.

## Introduction:

Over the past few decades, MPs have emerged as pervasive environmental contaminants^1, 2^, raising growing concern about their impact on human health^3^. Most existing studies linking MP to disease are correlative, focusing on MP detection in diseased tissues^4, 5, 6, 7^ or have noted altered tissue and cellular metabolism^8, 9, 10, 11, 12^. Of particular interest, is the impact of plastics on bone, as synthetic polymers are extensively used in orthopedic prostheses and implants, where long-term mechanical wear inevitably generates plastic debris at the bone-implant interface. Indeed, ultra-high molecular weight polyethylene (UHMWPE), a common material in joint replacements, has been shown to produce wear particles. These particles induced a catabolic, bone-resorbing phenotype in osteocytes without causing osteocyte death by directly introducing UHMWPE particles into the surgical site, although the particle size was unreported^13^.

While plastic wear particles from prosthetic materials are known to affect bone biology, more recent studies have been investigating the effects of MPs under non-iatrogenic conditions. Daily oral gastric gavage (OGG) of 5-μm polystyrene (PS)-MPs or modified PS-MPs for 28 days in mice impaired trabecular microarchitecture. Interestingly, PS-MPs accumulated in bones, potentially within the marrow compartment, although the exact localization was not determined^14^. Accumulation of 80 nm-sized PS nanoplastics (PSNP) were also found in bone tissue after daily OGG for 42 days, which resulted in impaired hematopoiesis in both the bone marrow and spleen, and reduced hematopoietic stem/progenitor cell populations. Mechanistically, PSNP exposure induced oxidative stress and activated oxeiptosis and senescence pathways, contributing to hematotoxicity^15^. Similar effects on the bone marrow were also observed after daily OGG of 5- and 10-μm sized for 42 days^16^. In rats, daily OGG of 1-μm PS MP impaired endochondral ossification by disrupting chondrocyte organization and inducing ER stress^17^. Another study showed intraperitoneal injection into rats caused skeletal toxicity^18^. These studies support a growing link between MP exposure and skeletal dysfunction primarily through systemic inflammation or marrow toxicity.

A major caveat with these prior *in vivo* studies is that they predominantly relied on bolus oral gavage, intraperitoneal injection, or delivery in drinking water ^14, 15, 16, 17, 18^. These exposure paradigms may not fully recapitulate typical dietary intake including regulated ingestion, normal digestive processing, and sustained luminal interactions within the gut. In contrast, dietary exposure more closely reflects human consumption by integrating MPs into physiological contexts that involve gastrointestinal (GI) motility, mucosal contact, and GI hormone release, all of which could influence MP uptake and downstream systemic effects. Despite this, few studies have investigated how MPs affect bone physiology when delivered through food, or how dietary composition modifies these effects. Diet might plausibly exacerbate or mitigate MP toxicity: high-fat, high-cholesterol diet could enhance MP absorption through impaired barrier integrity or enhanced lipid-mediated transport, whereas a high-fiber diet may limit MP uptake by strengthening mucosal defenses and reinforcing epithelial barrier function. Additionally, MPs have been shown to alter the gut microbiota and microbial metabolites^8, 19^. These interactions and effects are particularly relevant in light of growing evidence that MPs act as endocrine disruptors^20, 21, 22^. Additionally, enteroendocrine cells (EECs), the gut’s hormone-producing sentinels, which integrate luminal cues with systemic hormone signaling, may represent an early interface between ingested MPs and host metabolism and physiology. Although diet is a well-established regulator of the gut function, metabolism, and endocrine signaling, its role in shaping MP-induced toxicity, particularly with respect to skeletal health and underlying causal mechanisms, remains poorly understood.

**Figure 1.**
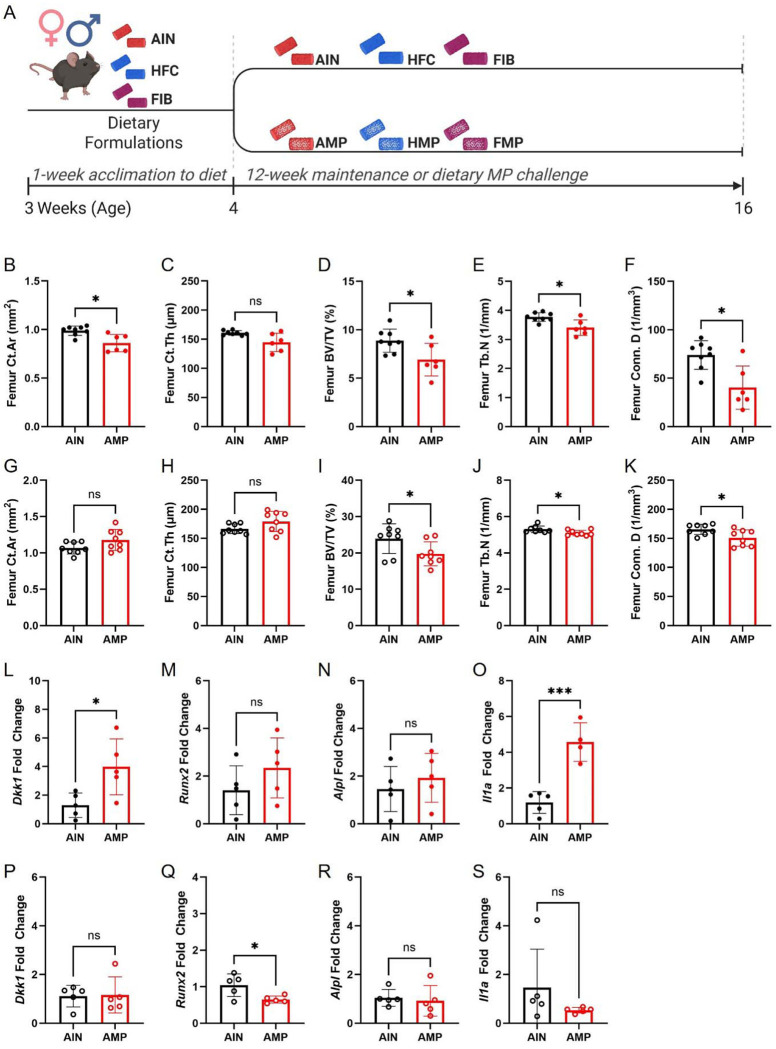
Dietary microplastic exposure disrupts bone microarchitecture in a sex-specific manner. **a,** Experimental design. Adolescent C57BL/6J female and male mice were acclimated at 3 weeks of age to one of three diets: modified AIN-93M basal diet (AIN), high-fat/high-cholesterol diet (HFC), or high-fiber diet (FIB). At 4 weeks of age, mice were maintained on these diets for 12 weeks with or without a physiologically relevant mixture of polystyrene microplastics (MPs; ~0.5, 1.0, 2.0, 3.0, and 5.0 μm; total dose 1.697 mg kg^−1^). Diets containing MPs are referred to as AMP (AIN + MPs), HMP (HFC + MPs), and FMP (FIB + MPs). **b-f,** Femoral μCT analysis in females (closed circles) showing reduced cortical area (Ct.Ar) and cortical thickness (Ct.Th) (**b,c**), together with decreased trabecular bone volume fraction (BV/TV) (**d**), trabecular number (Tb.N) (**e**), and connectivity density (Conn. D) (**f**) in AMP-fed mice relative to AIN controls. **g-k,** Femoral μCT analysis in males (open circles) demonstrating preserved cortical parameters (**g,h**) but reduced trabecular BV/TV (**i**), Tb.N (**j**), and Conn. D (**k**) in AMP-fed mice. **l-o,** Relative mRNA expression of *Dkk1* (**l**), *Runx2* (**m**), *Alpl* (**n**), and *Il1a* (**o**) in femoral bone from females. **p-s,** Relative mRNA expression of *Dkk1* (**p**), *Runx2* (**q**), *Alpl* (**r**), and *Il1a* (**s**) in femoral bone from males. Data are mean ± s.d.; dots represent individual mice. Statistical significance is indicated as *P < 0.05, ***P < 0.001; ns, not significant. See Extended Data Fig. 3 for vertebral μCT analyses.

Nevertheless, these studies do not capture the complex *in vivo* context in which bone homeostasis is regulated by endocrine, immune, and gut-derived signals. Accordingly, we set out to examine the influence of MPs in various purified formulas and the consequences they have on colonic and systemic tissues. Adolescent C57BL/6J (B6) female and male mice were fed one of three diets (detailed in **Extended Data Table 1**): (i) basal diets (modified AIN-93M, used as the base for all groups; herein called AIN), (ii) a high-fat, high-cholesterol diet (40% lard-base fat and 1.25% cholesterol; herein called HFC), or (iii) a high-fiber diet (10% added cellulose and inulin; herein called FIB) at 3 weeks of age to acclimate to dietary formulas. Female and male mice began dietary interventions at 4 weeks of age and were monitored for 12 weeks (Figure 1A). Each diet was provided with or without a physiologically relevant mixture of PS MPs (~0.49, 1.0, 1.9, 3.1, and 5.0 μm; ~1.7 mg/kg) (**Extended Data Table 2**). Diets with MPs are referred to as AMP (AIN with MPs), HMP (HFC with MPs), and FMP (FIB with MPs). Across all dietary groups, endpoint weight gain was unaltered between AIN and AMP groups, HFC and HMP groups, and FIB and FMP groups (**Extended Data Fig. 1A-F**). Similarly, food (**Extended Data Fig. 1G-L**) and energy (**Extended Data Fig. 1M-R**) intake remained consistent, allowing direct comparison of weight and body composition outcomes.

Interestingly, we observed significant diet- and sex-dependent effects of MP exposure on body composition, particularly lean and fat mass as determined by dual-energy X-ray absorptiometry (DEXA). Inspection of fat mass revealed only male FMP mice had increased fat mass compared to male FIB mice (**Extended Data Fig. 2A-F**). For lean mass (**Extended Data Fig. 2G-L**), female AMP mice had an increase in lean mass compared to female AIN mice while male FMP mice displayed decreased lean mass compared to male FIB mice. To assess whether chronic MP exposure influences skeletal health, DEXA was used to measure whole-body bone mineral density (BMD) and composition (**Extended Data Fig. 2M-R**). DEXA only revealed significantly reduced BMD in AMP-fed females compared to AIN controls, whereas no significant differences were detected in the other groups. These findings suggested that MP exposure may adversely affect skeletal integrity in a sex-specific manner.

Given the limited resolution of DEXA for distinguishing cortical from trabecular compartments, we next conducted high-resolution micro-computed tomography (μCT) of the femur and vertebrae to localize and characterize MP-related skeletal changes. μCT analysis demonstrated that chronic MP exposure selectively degraded trabecular microarchitecture in both sexes, with additional cortical deficits in females. In AMP-fed females, cortical area and total cross-sectional area were reduced without changes in cortical thickness, accompanied by lower trabecular BV/TV and trabecular number, greater trabecular separation, and reduced connectivity (Fig. 1B–F, **Extended Data Fig. 3A-C**). AMP-fed males also displayed substantial trabecular deterioration including reduced BV/TV, trabecular thickness, and trabecular number, with increased separation and diminished connectivity density; however, cortical structure remained unchanged (Fig. 1G–K, **Extended Data Fig. 3D-F**). To identify potential mechanisms, we profiled genes regulating bone formation (*Runx2, Alpl*), Wnt signaling (*Dkk1*), and inflammation (*Il1a*). In AMP females, combined cortical and trabecular bone loss was associated with an upregulation of *Dkk1* and *Il1a* (Fig. 1L–O), consistent with Wnt signaling inhibition and inflammation-driven osteoclast activation, suggesting enhanced bone resorption and suppressed bone formation. Notably, expression of *Runx2* and *Alpl* was unchanged in this group (Fig. 1L–O). In AMP males, where trabecular bone loss occurred without cortical involvement, *Runx2*, a key driver of osteoblast differentiation, was reduced (Fig. 1P–S), suggesting impaired bone formation is the dominant mechanism, while *Alpl, Dkk1* and *Il1a* genes remained unchanged (Fig. 1P–S). Spinal μCT revealed more modest, diet-dependent changes. AMP-fed females showed only reduced trabecular thickness (**Extended Data Fig. 3G-K**), whereas AMP-fed males exhibited increased trabecular thickness coupled with reduced connectivity density (**Extended Data Fig. 3L-P**).

In contrast, MP exposure under high-fat or high-fiber diets produced more limited femoral effects: HMP females showed no MP-related differences in cortical or trabecular structure compared to HFC females (**Extended Data Fig. 4A-H**), whereas HMP males exhibited reduced trabecular BV/TV and trabecular thickness with preserved cortical morphology (**Extended Data Fig. 4I-P**). HMP females showed no changes in *Dkk1, Runx2, Alpl*, or *Il1a* (**Extended Data Fig. 5A-D**), consistent with minimal skeletal changes. In contrast, HMP males exhibited reduced *Runx2* and *Alpl*, indicating impaired osteoblast differentiation and maturation, while markers of Wnt signaling (*Dkk1)* and inflammation (*Il1a)* remained unchanged (**Extended Data Fig. 5E-H**). For the high fiber groups, FMP females also showed no detectable impact of MP exposure (**Extended Data Fig. 6A-H**), while FMP-fed males displayed coordinated deficits across both compartments, including reduced cortical area, total area, and cortical thickness, together with lower trabecular BV/TV and trabecular thickness (**Extended Data Fig. 6I-P**). FMP-fed females and males had no significant changes in *Alpl, Runx2*, or *Dkk1* expression while *Il1a* was undetectable in both FIB and FMP samples (**Extended Data Fig. 7A-F**). These findings suggest that cortical and trabecular bone loss observed in FMP-fed males may be driven by alternative, as yet undefined mechanisms. No spine parameters were altered in HMP-females (**Extended Data Fig. 4Q-U**) or in FMP-fed females (**Extended Data Fig. 6Q-U**), while HMP-fed males displayed reduced BV/TV and trabecular thickness (**Extended Data Fig. 4V-Z**) and FMP-fed males showed decreased trabecular thickness with increased connectivity density (**Extended Data Fig. 6V-Z**). Collectively, these data demonstrate that chronic MP ingestion disrupts bone homeostasis in a sex- and diet-specific manner, with the trabecular compartment most consistently affected.

High-fat and high-fiber diets have been independently linked to altered bone physiology through mechanisms involving inflammation, nutrient absorption, and gut perturbations ^23, 24, 25^. We therefore assessed colonic health. Colon histopathology revealed uniformly low pathology scores across all diet and MP conditions (**Extended Data Fig. 8A-F, Extended Data Table 3**). Most animals displayed intact intestinal architecture with no evidence of inflammation, and when changes were present, they were limited to mild submucosal edema (combined score = 1) without polymorphonuclear infiltration, goblet cell depletion, or epithelial injury. These minimal findings occurred sporadically across all groups and did not cluster by diet type, MP exposure, or sex. Additional assessment of fecal lipocalin-2 (LCN2) and secretory IgA (sIgA) at baseline (1 week after acclimation to diet without MPs), 6 weeks and 12 weeks revealed no significant changes between groups (**Extended Data Fig. 8G-X**). Together, these findings indicate that MP-induced bone loss, particularly in animals fed the AMP diet, is not accompanied by overt colonic inflammation or epithelial injury, prompting investigation into alternative gut-derived signaling mechanisms.

**Figure 2.**
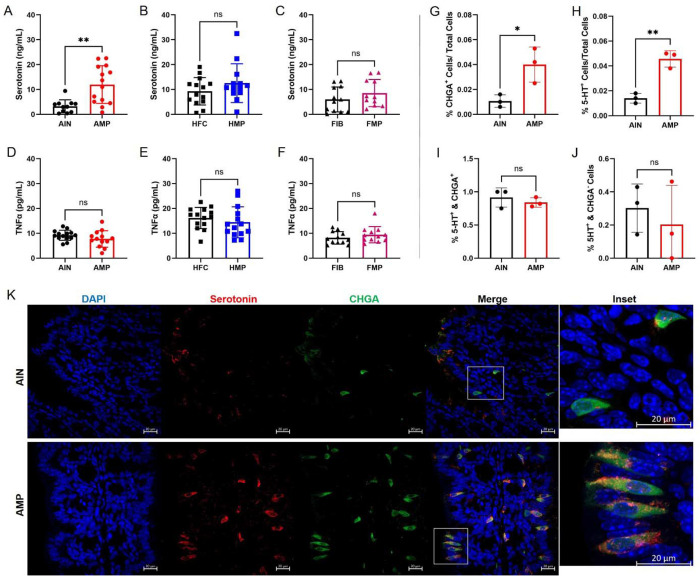
Dietary microplastic exposure enhances circulating serotonin and modulates enteroendocrine cell abundance without altering lineage identity. **a-c**, Targeted plasma hormone profiling showing increased circulating serotonin levels in AMP-fed mice compared with AIN controls (**a**), with no differences between HFC and HMP (**b**) or FIB and FMP (**c**) groups. **d-f**, Plasma TNFa levels for the corresponding dietary comparisons, showing no significant differences between MP-exposed and control groups. **g,h**, Quantification of colonic enteroendocrine cells by immunofluorescence demonstrating increased proportions of CHGA^+^ cells (**g**) and 5-HT^+^ cells (**h**) in AMP-fed mice relative to AIN controls. **i,j**, Quantitative analysis showing no change in the fraction of serotonergic cells within the CHGA^+^5-HT^+^ population (**i**) and no increase in CHGA^−^/5-HT^+^ cells (**j**), indicating preserved enterochromaffin lineage identity despite increased enteroendocrine cell abundance. **k**, Representative immunofluorescence images of proximal colon from AIN- and AMP-fed mice stained for nuclei (DAPI, blue), serotonin (5-HT, red), and CHGA (green). Merged images and inset highlight increased CHGA^+^ and 5-HT^+^ cells in AMP-fed mice. Scale bars, 20 μm. Data are shown as mean ± s.d.; dots represent individual mice. Statistical significance is indicated as *P < 0.05, **P < 0.01; ns, not significant. Additional hormone and cytokine measurements are shown in Extended Data Fig. 9.

With the absence of intestinal pathology or overt inflammatory responses, we next asked whether MP exposure alters gut-derived endocrine signals implicated in bone metabolism. Targeted plasma hormone profiling revealed a selective increase in circulating serotonin, a gut-derived signaling molecule with established inhibitory effects on osteoblast function^26, 27, 28^,, in AMP-fed mice compared with AIN controls (Fig. 2A). In contrast, serotonin concentrations did not differ between HFC and HMP groups or between FIB and FMP groups (Fig. 2B,C). Other EEC hormones were largely unaffected by MP exposure: Glucose-dependent Insulinotropic Polypeptide (GIP) and resistin did not differ between MP-exposed mice and diet-matched controls across any dietary condition, while Glucagon-like Peptide-1 (GLP-1) was modestly reduced only in FMP compared with FIB mice (**Extended Data Fig. 9A-I**). Increased circulating inflammatory cytokines have also been linked to skeletal regulation including TNFa, MCP-1 and IL-6^29, 30, 31, 32, 33, 34, 35^. Several studies have shown MP exposure increases TNFa levels^36, 37, 38, 39;^ however, TNFa did not differ between MP-exposed and control mice within any diet (Fig. 2D–F). Additionally, no change in MCP-1 was observed while IL-6 levels were only higher in HFC relative to HMP mice (**Extended Data Fig. 9J-O**). Notably, the selective elevation of serotonin, observed only under AMP conditions, coincided with the most pronounced bone loss observed in AMP-fed animals, supporting a potential endocrine contribution to MP-associated skeletal deficits.

To determine whether enhanced serotonergic signaling reflects intrinsic shifts in EEC differentiation, colonic organoids derived from AIN- and AMP-fed B6 mice were cultured and analyzed. Expression of enteroendocrine markers, including *Chga* (chromogranin A) and the serotonin biosynthetic enzyme *Tph1* (tryptophan hydroxylase 1), did not differ between AIN- and AMP-derived organoids in either stem cell-enriched or differentiated standard conditions (**Extended Data Fig. 10A-G**), indicating no sustained epithelial-intrinsic bias toward enteroendocrine lineage specification. We next examined EEC activity *in vivo*. Immunofluorescence staining of colonic tissue (Fig. 2G–K) from AIN- and AMP-fed mice revealed increased CHGA^+^ and 5-HT^+^ cells in AMP-fed animals (Fig. 2G,H). Further quantitative analysis demonstrated that the fraction of serotonergic cells within the CHGA^+^/5-HT^+^ and the CHGA^−^/5-HT^+^ population were unchanged suggesting that MP exposure increases the abundance and/or detectability of EECs while preserving enterochromaffin lineage identity (Fig. 2 I,J). These findings suggest proportional expansion or enhanced activity of the enteroendocrine compartment rather than selective skewing toward serotonergic differentiation. To further assess enterochromaffin cell-specific responses, we utilized *Piezo2*-GFP reporter mice^40^ where GFP is fused to the c-terminus of the endogenous Piezo2 protein and selectively labels a major subset of enterochromaffin cells^41^. AMP exposure increased serotonin (5-HT) immunoreactivity per cell without altering the number or overall GFP intensity of Piezo2^+^ cells (**Extended Data Fig. 10H-J**), further suggesting enhanced serotonergic output within an otherwise stable enterochromaffin population. Together, these data support a model in which dietary MP exposure augments enterochromaffin function with minimal changes in cell number detectable at the level of this reporter.

**Figure 3.**
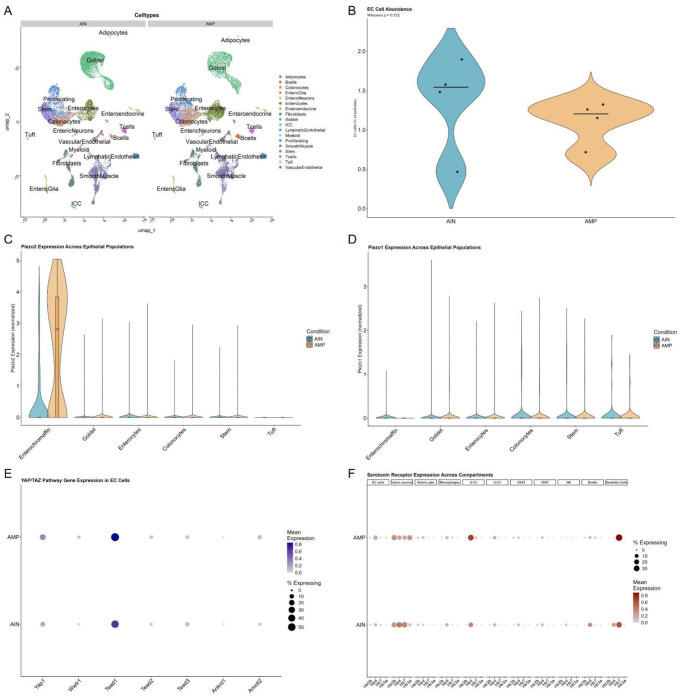
AMP exposure selectively modulates mechanosensory-associated transcripts in enterochromaffin cells without altering epithelial composition **a,** UMAP visualization of integrated single-nuclei RNA sequencing (snRNA-seq) data from colonic tissue of mice fed AIN or AMP diets (n = 4 biological replicates per group). Unsupervised clustering resolved major epithelial, immune, stromal, vascular, and neuromuscular compartments. Comparable cluster structure and cell identities between dietary conditions indicate preserved tissue architecture and minimal batch effects. **b**, Relative abundance of enterochromaffin (EC) cells across diets. Violin plots show the percentage of EC cells per biological replicate. No significant difference was detected between AIN and AMP groups (Wilcoxon test). **c**, *Piezo2* expression across epithelial populations. Violin plots show normalized single-cell expression stratified by cell type and diet. *Piezo2* transcripts were highly enriched in enterochromaffin cells and exhibited a rightward shift in AMP-fed mice, while remaining minimal in other epithelial populations. **d**, *Piezo1* expression across epithelial populations. Piezo1 transcripts were rare (<1% of EC cells) and did not differ between diets. **e,** Expression of YAP/TAZ pathway components in enterochromaffin cells. Dot plot showing mean expression (color intensity) and percentage of expressing cells (dot size) for *Yap1*, *Wwtr1* (Taz), *Tead1*, *Tead2*, *Tead3*, *Amot*, and *Amotl2*. Expression of core regulatory components was preserved across diets without coordinated induction of canonical YAP/TAZ target genes. **f**, Serotonin receptor (Htr family) expression across colonic compartments. Dot plot depicts mean expression and percent-expressing cells across epithelial, neural, and immune populations. Receptor expression patterns were largely conserved between diets; H*tr3a* expression in enteric neurons showed a directional increase in AMP-fed mice, consistent with enhanced neuronal serotonergic responsiveness in a subset of animals.

To define epithelial-intrinsic mechanisms at single-cell resolution, we performed single-nuclei RNA sequencing (snRNA-seq) on colonic tissue from AIN- and AMP-fed mice. Given the use of snRNA-seq, transcript detection reflects nuclear RNA abundance, which may underrepresent cytoplasmic mRNAs with rapid turnover; however, relative expression differences between dietary groups remain interpretable at the level of transcriptional regulation^42, 43, 44^. Unsupervised clustering resolved all major epithelial, immune, stromal, vascular, and neuromuscular compartments, with nearidentical cell identities and UMAP structure across dietary conditions (Fig. 3A; **Extended Data Fig. 11A**), arguing against widespread transcriptional disruption. Epithelial cell composition was largely preserved, with no evidence for expansion of enterochromaffin cells (Fig. 3B; **Extended Data Fig. 11B**), consistent with organoid and reporter analyses. Enterochromaffin cells were robustly identified by expression of canonical markers (*Tph1, Chga, Tac1*), and differential abundance analysis indicated minimal changes across conditions. A modest reduction in goblet cells was observed under AMP conditions (**Extended Data Fig. 11B**), a change unlikely to account for increased serotonin given the absence of inflammatory gene signatures (**Extended Data Fig. 12A**) or histopathologic evidence of barrier disruption (**Extended Data Fig. 7**).

Within this preserved epithelial landscape, enterochromaffin cells exhibited a focused transcriptional shift consistent with altered mechanosensory responsiveness. *Piezo2* transcripts were highly enriched in enterochromaffin cells and displayed a rightward shift in expression distribution under AMP conditions, while remaining minimal across other epithelial lineages (Fig. 3C); however, pseudo-bulk aggregation at the level of individual animals revealed substantial inter-animal variability, indicating that the magnitude of induction differed across biological replicates. The directionally consistent shift across animals, despite variable magnitude, suggests diet-responsive regulation with heterogeneous penetrance rather than stochastic noise. *Piezo1* expression was rare in enterochromaffin cells (<1%) and unchanged between diets (Fig. 3D). Consistent with the reporter model design, increased Piezo2 production would be expected to proportionally increase GFP signal. However, anti-GFP immunofluorescence revealed no overt change in reporter intensity within enterochromaffin cells under AMP conditions (Fig. 2I). Because GFP reports total fused protein abundance but not subcellular localization or channel activity, post-translational mechanisms including altered membrane trafficking, gating, or protein stability could enhance Piezo2 function without changing total GFP-detectable signal^45, 46, 47^. Notably, *Tph1* expression did not differ between diets (**Extended Data Fig. 11C**), indicating that increased serotonin production is unlikely to reflect transcriptional induction of the serotonergic biosynthetic machinery. Together, these findings support a model in which dietary MP exposure enhances enterochromaffin secretory output through activitydependent modulation of mechanosensory pathways, with proportional changes in EEC abundance or hormone content but without evidence of lineage reprogramming or induction of serotonergic gene expression programs.

Further analysis revealed enterochromaffin cells expressed the canonical transcriptional regulators *Yap1* and *Tead1* under both dietary conditions, consistent with a YAP/TAZ regulatory context capable of responding to mechanical cues (Fig. 3E); however, we did not observe coordinated induction of canonical YAP/TAZ target genes in AMP, suggesting engagement of mechanosensory signaling without widespread transcriptional reprogramming. Extending these observations, the mechanosensory-associated genes *Robo2* and its ligand *Slit3* exhibited increased expression across colonocytes, goblet cells, and enterochromaffin cells (**Extended Data Fig. 12B**), further supporting modulation of epithelial mechanosensory pathways in response to AMP exposure. Taken together, the data support modulation of mechanosensory responsiveness at the level of channel sensitivity or signaling efficiency rather than broad transcriptional remodeling of the enterochromaffin lineage.

We also examined expression of serotonin receptor genes (Htr family) across epithelial, neural, and immune compartments to assess potential downstream responsiveness within the colonic microenvironment (Fig. 3F). Receptor expression patterns were largely preserved between diets; however, *Htr3a* transcripts in enteric neurons displayed a directionally consistent increase following AMP exposure. Single-nuclei differential expression analysis identified enrichment of *Htr3a* expression in AMP-exposed mice, whereas pseudo-bulk aggregation at the level of individual animals revealed a similar fold change that did not reach statistical significance, reflecting inter-animal variability. These data suggest enhanced neuronal sensitivity to serotonergic input in a subset of animals. Collectively, these findings support engagement of mechanosensory-associated pathways within enterochromaffin cells as a plausible epithelial mechanism linking dietary MP exposure to elevated peripheral serotonin and downstream skeletal effects, occurring in the absence of inflammation, epithelial restructuring, or widespread receptor reprogramming.

**Figure 4.**
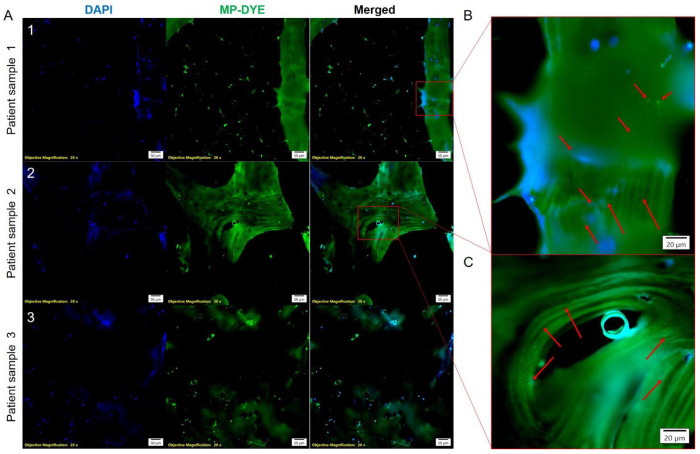
Microplastic (MP) detection in human distal femur tissue from donors without orthopedic prostheses. **a**, Representative fluorescence images of histological sections from distal femurs of three independent human donors (Patients 1-3; donor information in Extended Data Table 1) stained with DAPI (blue) to label nuclei and a conjugated polymer nanoparticle-based MP dye (MP-DYE, green). Merged images demonstrate discrete MP-positive signals within intact bone tissue. Objective magnification: 20X. Scale bars, 50 μm. **b-c**, Higher-magnification views of boxed regions in (**a**) highlighting (red arrows) irregular MP-positive signals (green) embedded within the bone matrix. Scale bars, 20 μm.

Lastly, because orthopedic prostheses and implants can generate plastic debris at the bone-implant interface, we sought to determine whether MPs are detectable in human skeletal tissue in individuals without prostheses. Thus, we analyzed histological sections of the human distal femur (from a commercial source, donor information in **Extended Data Table 4**). Building on recent reports of MP in human skeletal tissues identified by microphotography and Raman spectroscopy^18^, we applied a conjugated polymer nanoparticle-based dye, previously validated for stable, long-term microplastic detection^48^, to visualize MP-positive signals in intact human distal femur sections. Using this approach, MP-positive signals were detected in distal femurs from three independent human donors (Figure 4A–C). The specificity of this staining approach was supported by parallel analysis of liver tissue from mice maintained on control diets or MP-supplemented diets, in which polymer-positive signals were readily detected in MP-exposed animals (**Extended Data Fig. 13**). The detection of polymer signals in mineralized human tissue suggests that MPs can persist and accumulate in the skeleton, raising the possibility that environmental MP exposure is an unrecognized risk factor for bone fragility. Consistent with this possibility, *in vitro* studies have demonstrated that PS MPs impair osteogenic differentiation and induce senescence in MC3T3-E1 pre-osteoblasts^14^. To examine the direct effects of MPs and serotonin on human bone-forming cells, primary human osteoblasts from three independent donors (donor information in **Extended Data Table 5**) were cultured for 9 days in the presence of 1- or 5-μm PS MP (1 μg mL^−1^, **Extended Data Table 2**) or serotonin (10 or 50 ng mL^−1^), followed by washing and induction of mineralization (**Extended Data Fig. 14A**). In osteoblasts derived from two donors in their mid-70s, transient exposure to PS MPs resulted in a marked and sustained reduction in calcium deposition compared with untreated, donor-matched controls (**Extended Data Fig. 14B**). In these same donors, serotonin exposure also impaired mineralization, although sensitivity varied, with suppression observed at 50 ng mL^−1^ in one donor and 10 ng mL^−1^ in the other. In contrast, osteoblasts from a third donor in their mid-60s exhibited preserved mineralization following MP or serotonin exposure, indicating inter-individual variability in osteoblast sensitivity to serotonergic and microplastic perturbation. Together, these findings demonstrate that microplastics can persist in human bone tissue and disrupt osteoblast mineralization capacity in a donor-dependent manner.

Overall, our findings position MPs not merely as inert environmental contaminants but as bioactive dietary constituents capable of altering metabolic physiology in part through mechanosensory pathways. Rather than inducing overt intestinal injury or systemic inflammation, chronic dietary MP exposure preferentially perturbs gut-endocrine signaling. Across multiple dietary contexts, MP ingestion disrupted skeletal homeostasis in a sex- and diet-dependent manner, with the trabecular compartment most consistently affected. Under basal purified diet conditions, MP exposure was associated with enhanced gut-derived serotonergic signaling, independent of inflammatory cytokine induction. Although enterochromaffin cells are the predominant source of peripheral serotonin, enteroendocrine populations exhibit functional heterogeneity and hormone co-expression, raising the possibility that MP exposure enhances serotonergic output through activation or reprogramming of existing enteroendocrine cells rather than expansion of a discrete sub-lineage. This finding together with modest shifts in goblet cell abundance in the absence of inflammatory pathology suggests that subtle changes in mucus barrier dynamics may enhance epithelial exposure to luminal MPs facilitating mechanosensory engagement of enterochromaffin cells. Single-nuclei profiling further contextualized these effects by revealing discrete shifts in mechanosensory-associated pathways within enterochromaffin cells and potential downstream serotonergic targets across epithelial, enteric neuronal, and immune compartments, without evidence of generalized inflammatory activation. Together, these findings identify the gut endocrine system as a mechanosensory-sensitive interface through which ingested MPs may influence systemic physiology. Future studies employing pharmacologic or genetic modulation of peripheral serotonergic signaling will be required to determine whether gut-derived serotonin causally mediates the skeletal consequences of chronic MP exposure. Although classical systemic markers of bone turnover such as P1NP, osteocalcin, and CTX were not measured, and dynamic histomorphometry was not performed, the transcriptional changes observed in bone tissue are consistent with MP exposure influencing both osteoblast and osteoclast regulatory pathways. Notably, expression of *Tnfs11* (RANKL) in bone was unchanged across AIN and HFD dietary conditions (data not shown), suggesting that the skeletal phenotypes observed here are unlikely to be explained solely by alterations in canonical osteoclastogenic signaling. Instead, increased expression of inflammatory mediators and Wnt signaling antagonists in AMP females is consistent with enhanced bone resorption, whereas reduced Runx2 expression in AMP males suggests impaired osteoblast differentiation. Future studies integrating serum bone turnover markers and dynamic histomorphometric analyses will be required to define how MP exposure alters bone remodeling dynamics.

These findings extend the concept of dietary constituents beyond nutrients and bioactive metabolites to include synthetic particulate materials capable of modulating endocrine and metabolic physiology. The detection of polymer signals in intact human femoral tissue, together with diet-dependent skeletal and endocrine effects *in vivo*, suggests that chronic environmental microplastic exposure may represent an underappreciated modifier of bone health. Although our study does not establish causality in humans, it identifies a gut-bone endocrine axis responsive to dietary microplastics and highlights mechanosensory enterochromaffin signaling as a potential interface between environmental materials and host physiology. Whether similar serotonergic responses occur in humans and how dietary composition modulates skeletal vulnerability warrant further investigation.

## Supplementary Material

Supplement 1

## Data Availability

All primary data associated with this study are present in the paper or the Supplementary Materials.

## References

[1] Sajedi S., An C. & Chen Z. Unveiling the hidden chronic health risks of nano- and microplastics in single-use plastic water bottles: A review. J Hazard Mater 495, 138948 (2025). 40555020 10.1016/j.jhazmat.2025.138948

[2] Zimmermann L. Food contact articles as source of micro- and nanoplastics: a systematic evidence map. NPJ Sci Food 9, 111 (2025). 40562768 10.1038/s41538-025-00470-3 PMC12198380

[3] Lamoree M.H. Health impacts of microplastic and nanoplastic exposure. Nat Med 31, 2873–2887 (2025). 40935856 10.1038/s41591-025-03902-5

[4] Wu F. Microplastic accumulation in fibrotic intestinal tissue and mesenteric adipose tissue in Crohn’s disease patients. Environ Res 271, 121077 (2025). 39947377 10.1016/j.envres.2025.121077

[5] Horvatits T. Microplastics detected in cirrhotic liver tissue. EBioMedicine 82, 104147 (2022). 35835713 10.1016/j.ebiom.2022.104147 PMC9386716

[6] Marfella R. Microplastics and Nanoplastics in Atheromas and Cardiovascular Events. N Engl J Med 390, 900–910 (2024). 38446676 10.1056/NEJMoa2309822 PMC11009876

[7] Nihart AJ. Bioaccumulation of microplastics in decedent human brains. Nat Med 31, 1114–1119 (2025). 39901044 10.1038/s41591-024-03453-1 PMC12003191

[8] Garcia M.M. In Vivo Tissue Distribution of Polystyrene or Mixed Polymer Microspheres and Metabolomic Analysis after Oral Exposure in Mice. Environ Health Perspect 132, 47005 (2024). 38598326 10.1289/EHP13435 PMC11005960

[9] Cheng W. Integrated transcriptomics and metabolomics to explore the varied hepatic toxicity induced by aged- and pristine-microplastics: in vivo and human-originated liver organoids-based in vitro study. Environ Res 280,121820 (2025). 40378997 10.1016/j.envres.2025.121820

[10] Song Z., Wu H., Fang X., Feng X. & Zhou L. The cardiovascular toxicity of polystyrene microplastics in rats: based on untargeted metabolomics analysis. Front Pharmacol 15, 1336369 (2024). 38799170 10.3389/fphar.2024.1336369 PMC11127592

[11] Merkley S.D. Polystyrene microplastics induce an immunometabolic active state in macrophages. Cell Biol Toxicol 38, 31–41 (2022). 34021430 10.1007/s10565-021-09616-x PMC8606615

[12] Lin S. Metabolomics Reveal Nanoplastic-lnduced Mitochondrial Damage in Human Liver and Lung Cells. Environ Sci Technol 56, 12483–12493 (2022). 36005547 10.1021/acs.est.2c03980 PMC9454251

[13] Ormsby R.T. Evidence that osteocyte perilacunar remodelling contributes to polyethylene wear particle induced osteolysis. Acta Biomater 33, 242–251 (2016). 26796208 10.1016/j.actbio.2016.01.016

[14] Pan C. Polystyrene microplastics arrest skeletal growth in puberty through accelerating osteoblast senescence. Environ Pollut 322,121217 (2023). 36746288 10.1016/j.envpol.2023.121217

[15] Guo X. Polystyrene nanoplastics induce haematotoxicity with cell oxeiptosis and senescence involved in C57BL/6J mice. Environ Toxicol 38, 2487–2498 (2023). 37466197 10.1002/tox.23886

[16] Jing J. Polystyrene micro-/nanoplastics induced hematopoietic damages via the crosstalk of gut microbiota, metabolites, and cytokines. Environ Int 161,107131 (2022). 35149446 10.1016/j.envint.2022.107131

[17] Zhang Q. Polystyrene microplastic-induced endoplasmic reticulum stress contributes to growth plate endochondral ossification disorder in young rat. Environ Toxicol 39, 3314–3329 (2024). 38440912 10.1002/tox.24182

[18] Yang Q. Microplastics in human skeletal tissues: Presence, distribution and health implications. Environ Int 196, 109316 (2025). 39946929 10.1016/j.envint.2025.109316

[19] Kim KJ. In vivo exposure of mixed microplastic particles in mice and its impacts on the murine gut microbiome and metabolome. ToxicolSci (2025). 10.1093/toxsci/kfaf145 PMC12863214 41143690

[20] Ye T. Synergistic endocrine disruption and cellular toxicity of polyethylene microplastics and bisphenol A in MLTC-1 cells and zebrafish. Sci Rep 15, 10752 (2025). 40155689 10.1038/s41598-025-94902-5 PMC11953243

[21] Lin W. Polystyrene microplastics enhance the microcystin-LR-induced gonadal damage and reproductive endocrine disruption in zebrafish. Sci Total Environ 876, 162664 (2023). 36894083 10.1016/j.scitotenv.2023.162664

[22] Jahedi F. Nano and microplastics: unveiling their profound impact on endocrine health. Toxicol Meeh Methods 35, 865–893 (2025). 10.1080/15376516.2025.2509745 40432394

[23] Wang N., Tong X. & Li Y.K. High-fat diet, intestinal microecology and bone loss. Nutr Metab (Land) 22, 117 (2025). 10.1186/s12986-025-01013-z PMC12506283 41063146

[24] Zhu R. High-Fat Diet Increases Bone Loss by Inducing Ferroptosis in Osteoblasts. Stem Cells Int 2022, 9359429 (2022). 36277036 10.1155/2022/9359429 PMC9586793

[25] Wang R. Dietary Fiber Intake Improves Osteoporosis Caused by Chronic Lead Exposure by Restoring the Gut-Bone Axis. Nutrients 17 (2025). 10.3390/nu17091513 PMC12073446 40362820

[26] Yadav V.K. Lrp5 controls bone formation by inhibiting serotonin synthesis in the duodenum. Cell 135, 825–837 (2008). 19041748 10.1016/j.cell.2008.09.059 PMC2614332

[27] Karsenty G. & Yadav V.K. Regulation of bone mass by serotonin: molecular biology and therapeutic implications. Annu Rev Med 62, 323–331 (2011). 21073335 10.1146/annurev-med-090710-133426

[28] Kode A. Lrp5 regulation of bone mass and serotonin synthesis in the gut. Nat Med 20, 1228–1229 (2014). 25375916 10.1038/nm.3698

[29] Manolagas S.C. Role of cytokines in bone resorption. Bone 17, 63S–67S (1995). 8579900 10.1016/8756-3282(95)00180-l

[30] Blaschke M. IL-6, IL-lbeta, and TNF-alpha only in combination influence the osteoporotic phenotype in Crohn’s patients via bone formation and bone resorption. Adv Clin Exp Med 27, 45–56 (2018). 29521042 10.17219/acem/67561

[31] Kurokouchi K. TNF-alpha increases expression of IL-6 and ICAM-1 genes through activation of NF-kappaB in osteoblast-like ROS17/2.8 cells. J Bone Miner Res 13, 1290–1299 (1998). 9718198 10.1359/jbmr.1998.13.8.1290

[32] Mulholland B.S., Forwood M.R. & Morrison N.A. Monocyte Chemoattractant Protein-1 (MCP-1/CCL2) Drives Activation of Bone Remodelling and Skeletal Metastasis. Curr Osteoporos Rep 17, 538–547 (2019). 31713180 10.1007/s11914-019-00545-7 PMC6944672

[33] Siddiqui J.A. & Partridge N.C. CCL2/Monocyte Chemoattractant Protein 1 and Parathyroid Hormone Action on Bone. Front Endocrinol (Lausanne) 8, 49 (2017). 28424660 10.3389/fendo.2017.00049 PMC5372820

[34] Siddiqui J.A. Catabolic Effects of Human PTH (1-34) on Bone: Requirement of Monocyte Chemoattractant Protein-1 in Murine Model of Hyperparathyroidism. SciRep 7, 15300 (2017). 10.1038/s41598-017-15563-7 PMC5681546 29127344

[35] Lorenzo J. Interactions between immune and bone cells: new insights with many remaining questions. J Clin Invest 106, 749–752 (2000). 10995785 10.1172/JCI11089 PMC381401

[36] Djouina M. Oral exposure to polyethylene microplastics alters gut morphology, immune response, and microbiota composition in mice. Environ Res 212,113230 (2022). 35398082 10.1016/j.envres.2022.113230

[37] Gaspar L., Bartman S., Coppotelli G. & Ross J.M. Acute Exposure to Microplastics Induced Changes in Behavior and Inflammation in Young and Old Mice. IntJ Mol Sci 24 (2023). 10.3390/ijms241512308 PMC10418951 37569681

[38] Moon H. Microplastic exposure linked to accelerated aging and impaired adipogenesis in fat cells. Sci Rep 14, 23920 (2024). 39397046 10.1038/s41598-024-74892-6 PMC11471870

[39] Wang Z. Transfer toxicity of polystyrene microplastics in vivo: Multi-organ crosstalk. Environ Int 202, 109604 (2025). 40532535 10.1016/j.envint.2025.109604

[40] Woo S.H. Piezo2 is required for Merkel-cell mechanotransduction. Nature 509, 622–626 (2014). 24717433 10.1038/nature13251 PMC4039622

[41] Alcaino C. A population of gut epithelial enterochromaffin cells is mechanosensitive and requires Piezo2 to convert force into serotonin release. Proc Natl Acad Sci USA 115, E7632–E7641 (2018). 30037999 10.1073/pnas.1804938115 PMC6094143

[42] Habib N. Massively parallel single-nucleus RNA-seq with DroNc-seq. Nat Methods 14, 955–958(2017). 28846088 10.1038/nmeth.4407 PMC5623139

[43] Bakken T.E. Single-nucleus and single-cell transcriptomes compared in matched cortical cell types. PLoS One 13, e0209648 (2018). 30586455 10.1371/journal.pone.0209648 PMC6306246

[44] Slyper M. A single-cell and single-nucleus RNA-Seq toolbox for fresh and frozen human tumors. Nat Med 26, 792–802 (2020). 32405060 10.1038/s41591-020-0844-1 PMC7220853

[45] Snapp E.L. Fluorescent proteins: a cell biologis’s user guide. Trends Cell Biol 19, 649–655 (2009). 19819147 10.1016/j.tcb.2009.08.002 PMC2784028

[46] Oribamise E.I., Li Q. & Garry M.G. Coordinated regulation of PIEZ02 by alternative splicing, post-translational modification, membrane trafficking and protein partners. J Physiol (2026). 10.1113/JP290592 41631962

[47] Jia Z., Ikeda R., Ling J., Viatchenko-Karpinski V. & Gu J.G. Regulation of Piezo2 Mechanotransduction by Static Plasma Membrane Tension in Primary Afferent Neurons. J Biol Chern 291, 9087–9104 (2016). 10.1074/jbc.M115.692384 PMC4861477 26929410

[48] Peace H. Stable staining of microplastics using conjugated polymer nanoparticles. Environmental Science: Nano 12, 2229–2233 (2025).

