# Supplementary material for: Dietary Microplastics Engage Gut Mechanosensory-Endocrine Signaling to Disrupt Bone Homeostasis
Source: bioRxiv. 2026 Apr 7:2026.04.03.716216. Preprint. [Version 1] doi: 10.64898/2026.04.03.716216 (PMC13081867; doi:10.64898/2026.04.03.716216)
Supplement: Supplement 1 [file media-1.docx]

**Supplementary Information**

**Dietary Microplastics Engage Gut Mechanosensory-Endocrine Signaling to Disrupt Bone Homeostasis**

# Aaron S. Romero, Sumira Phatak et al.,

**Contents: Materials and methods, supplementary figures legends, supplementary figures, and references.**

**Materials and methods**

**Animals.** Male and female C57BL/6J (Strain #000664) were purchased from The Jackson Laboratory (JAX) at three weeks of age. Additionally, male and female B6(SJL)-*Piezo2*^tm1.1(cre)Apat^/J (Strain #027719) PIEZO2-GFP reporter mice were purchased from JAX between 5-7 weeks of age. Animals were maintained at constant temperature (20-24°C), relative humidity (30%-60%), and 12-hlight/dark cycle throughout the study. Animals were provided with multiple diets and water ad libitum. All experiments were approved by the Institutional Animal Care and Use Committee of the University of New Mexico Health Sciences Center, in accordance with the National Institutes of Health guidelines for use of live animals. The University of New Mexico Health Sciences Center is accredited by the American Association of Accreditation of Laboratory Animal Care.

**Dietary Exposure to Microplastics.** Mice were exposed to a mixture of polystyrene microspheres via their diet over a 12-wk period with either 0% or 1.697% polystyrene microspheres (Magsphere, Pasadena, CA). Exposure was based on an estimated average human intake of 5 grams plastics ingested per week^1^. Five sizes of polystyrene microspheres were utilized by mass: 0.49 µm (Magsphere, catalog no. PSFB500NM), 1.0 µm (Magsphere, catalog no. PSMG001UM), 1.9 µm (Magsphere, catalog no. PSOF002UM), 3.1 µm (Magsphere, catalog no. PSFY003UM), and 5.0 µm (Magsphere, catalog no. PSFR005UM) (**Extended Data Table 2**). Base diets included AIN-93M (AIN, Product no. D10012MO), 40% fat from lard plus 1.25% added cholesterol (HFC, Product no. D24020804), and 10% added inulin high-fiber (FIB, Product no. D24020806) purified formulas (Research Diets Inc., New Brunswick, NJ) (**Extended Data Table 1**). Microspheres were washed 10 times using sterile, deionized water to remove surfactant and sodium azide prior to formulation with AIN (AMP, Product no. D24020803), HFC (HMP, Product no. D24020805), and FIB (FMP, Product no. D24020807) diets (**Extended Data Table 1**). Mice were weighed weekly and food intake was tracked biweekly throughout the study. After 12 weeks, mice were euthanized using isoﬂurane and exsanguinated, then systemically perfused with ice cold saline to ensure removal of blood from major organs.

**Cells and Tissue Preparation.** Upon necropsy, tissues were weighed, measured, sectioned out for various procedures, and flash frozen. Samples collected for downstream analysis included colon, liver, plasma, spinal cords, femurs, and stool. These samples were either flash frozen in liquid nitrogen and stored in -80C, fixed with formalin, or put into 70% ethanol (EtOH). Blood was collected via cardiac puncture and collected in the BD P800 blood collection tube containing proprietary cocktail of protease, esterase and DPP-IV Inhibitors (BD Biosciences, catalog no. 366420) for plasma isolation. Plasma was used for serotonin (5-HT) detection via ELISA (Abnova, Catalog no. KA1894) or hormone (Amylin; C-Peptide; Ghrelin; GIP; GLP-1 (total); Glucagon; Insulin; Leptin; PP; PYY; Resistin; and Secretin) and cytokine (IL-6, MCP-1/CCL2; TNFα) detection by a MILLIPLEX mouse metabolic hormone expanded panel multiplex assay (Millipore, catalog no. MMHE-44K). Stool samples collected from weeks 0, 3, 6, 9, and 12 were processed and normalized to 90mg/mL using PBS + 0.1% TWEEN to analyze inflammatory markers of the colon such as Lipocalin-2 (LCN2)^2^ using R&D Systems Quantikine ELISA Mouse Lipocalin-2/NGAL Immunoassay (Catalog no. MLCN20) and secretory IgA (sIgA) from Bio-Techne Mouse Secretory IgA ELISA Kit (colorimetric) (Catalog no. NBP3-11824). A section of the proximal colon was stored in 10% formalin for 48 hours, then washed and stored in 70% EtOH prior to processing for tissue sectioning. Histopathological evaluation was performed on mouse colon tissue. Formalin-fixed, paraffin-embedded (FFPE) sections of proximal colon were prepared at the University of New Mexico Comprehensive Cancer Center (UNMCCC) Human Tissue Repository (HTR) Core Facility. Sections (5 μm) were cut from blocks fixed in 10% neutral-buffered formalin and stained with hematoxylin and eosin (H&E) using standard protocols. The degree of intestinal inflammation scoring was performed based on the guidelines provided by Erben et al^3^. Colon sections were scored for histopathological features of colitis based on established criteria, including: submucosal edema (0–3), polymorphonuclear leukocyte (PMN) infiltration (0–3), goblet cell depletion (0–3), and epithelial integrity (0–3) (**Extended Data Table** **3**).

**Micro-CT Analysis.** Spinal cords and femurs were collected from all mice to measure cortical area and thickness as well as trabecular volume and separation using a micro-CT (µCT) scanner. Indices of trabecular and cortical bone volume and structure were measured in the spine (excised 5th lumbar spine body) and the femur, respectively, using a Scanco mCT‐40 scanner (Scanco Medical, Bassersdorf, Switzerland). µCT scanning and analysis was performed as reported previously. Briefly, trabecular and cortical bone regions were evaluated using isotropic 12‐mm voxels. For the vertebral trabecular region, we evaluated 250 transverse CT slices between the cranial and caudal end plates, excluding 100mm near each end plate. For the femoral trabecular region, we analyzed 100 slices from the 50 slices under the distal growth plate. Femoral cortical bone was assessed using 50 continuous CT slides located at the femoral midshaft.

**Generation of intestinal organoids**. Intestinal organoid media was comprised of Advanced Dulbecco’s modified Eagle medium/Ham’s F-12 (ThermoFisher, Waltham, MA), 100 U/mL penicillin/streptomycin (Quality Biological, Gaithersburg, MD), WNT surrogate-Fc fusion protein (ImmunoPrecise Antibodies Ltd, Utrecht, The Netherlands) 15% v/v R-spondin1 conditioned medium (cell line kindly provided by Calvin Kuo, Stanford University), 10% v/v Noggin conditioned medium (cell line kindly provided by Gijs van den Brink, Tytgat Institute for Liver and Intestinal Research), 1X B27 supplement (ThermoFisher), 10 mM HEPES (ThermoFisher), 1X GlutaMAX (ThermoFisher), 1mM N-acetylcysteine (MilliporeSigma), 50 ng/mL human epidermal growth factor (ThermoFisher), 10 nM [Leu-15] gastrin (AnaSpec, Fremont, CA), 500 nM A83-01 (Tocris, Bristol, United Kingdom), 10 μM SB202190 (MilliporeSigma), 100 mg/mL primocin (InvivoGen, San Diego, CA). Base media, for differentiation of organoids, had the same composition of media but lacked WNT surrogate Fc fusion protein, Rspo-1 and SB202190. Colonic crypt isolation and colonic organoid generation were prepared as previously reported ^4, 5, 6, 7^. Isolated crypts from proximal mouse colon were resuspended in Matrigel (Corning) and 25 µL droplets were plated in a 24-well tissue culture plate (Corning). After polymerization at 37°C, 500µL of organoid expansion media was added for 2 d. After 2 d, the organoid expansion media was replaced every other day. Colonic organoids were passaged every 2-3 days by harvesting in Cultrex Organoid Harvesting Solution (Bio-Techne, Minneapolis, MN) at 4°C with shaking for 45 min, as previously described ^6, 7^. All colonic organoids cultures were maintained at 37°C and 5% CO2. Unless noted, colonoid lines have been passaged >30 times. Colonic organoids were harvested from Matrigel using Cultrex Organoid Harvesting Solution as previously described ^6, 7^.

**RNA Isolation, Quantification, and RT-qPCR**. RNA Isolation was performed on C57BL/6J mice femurs following shaving of muscle off bone, mashing of the bone with a mortar and pestle, and being put into TRIzol Reagent (Thermofisher) then using the RNA Purelink Minikit (Thermofisher) according to the manufacturer’s protocol. RNA isolation was performed using the RNA Purelink Minikit (Invitrogen) according to the manufacturer’s protocols on colonic organoids which were harvested from Matrigel using Cultrex Organoid Harvesting Solution as previously described^7^. RNA was quantified using a Nanodrop2000 and all samples yielded a 260/280 of 2 ± 0.15. cDNA synthesis was performed using a BioRad MyCycler Thermal Cycler and SuperScript™ IV VILO™ Master Mix (Thermofisher) and normalizing to the lowest yielding RNA concentrations. TaqMan™ Universal PCR Master Mix (Thermofisher) was used to perform qPCR using primers listed in **Extended Data Table 6**. Using 384 well plates, qPCR was performed on a CFX384 Touch Real-Time PCR Detection System.

**Microscopy, Image analysis, Immunofluorescence and Histology.** Unstained slides of paraffin-embedded colon from mice or human femurs (**Extended Data Table 4**, OriGene Technologies, Inc) were stained with antibodies or dyes listed (**Extended Data Table 7**) using antigen retrieval. Briefly, slides were baked for 2 hours and allowed to cool for 30 mins. Slides were subsequently washed with xylene 2x and EtOH 5x at 100, 95, 80, 70, and 50% concentrations. Slides were then placed in 10mM of sodium citrate buffer (2.94g/1L of H2O) and microwaved on high for 5 minutes following by cooling at RT for 10 minutes 2x. After the second buffer placement, slides were placed in fresh sodium citrate buffer for 20 minutes to cool. Slides were then immersed in 0.85% NaCl for 5 minutes, dipped in ddH2O 3x and washed with 0.01M Dulbecco’s PBS 2x. Slides then had PAP pen circling and were allowed to dry for 15 minutes and washed with PBS 2x. Slides were then incubated with Image-iT FX Signal Enhancer in a H2O humidity chamber for 30 mins, and placed in primary antibody overnight in perm-block buffer (0.1% saponin, 2% BSA, 15% FBS, brought up in 1X PBS). After overnight incubation, slides were washed with 0.01M PBS 3x and incubated with secondary antibody for 2 hours in immuno-block buffer. Slides were washed 3x with PBS, 1x with ddH2O, and dried for 30 minutes. Slides then had a coverslip mounted with fructose-glycerol solution (2.5M fructose-60% glycerol stock). Immunofluorescence images were captured utilizing a Zeiss LSM800 Confocal Microscope at 40X objectives. Images were processed using Zeiss ZEN (Version 3.7) software. Imaging of H&E-stained and MP-Dye tissue sections was performed using the Evident APEXVIEW APX100 Benchtop Fluorescence Microscope, which supports both brightfield and fluorescence imaging with appropriate filter sets (e.g., DAPI, FITC, TRITC). Acquisition parameters in the cellSens APEX software were standardized across all samples, and images were exported as high-resolution TIFF files. All primary and secondary antibodies used for confocal staining are listed in **Extended Data Table 7**.

**Single-nuclei RNA Sequencing and Bioinformatic Analysis**. Colonic tissue from female mice maintained on AIN or AMP diets (n = 4 biological replicates per group) was enzymatically dissociated using the Chromium Nuclei Isolation kit with RNase Inhibitor (Catalog no. PN-1000494, 10x Genomics) to generate single-nuclei suspensions. Single-nuclei RNAseq libraries were prepared using Chromium Next GEM 3’ Reagent Kit v3.1 following manufacturer’s instructions (protocol CG000315 Rev F, 10x Genomics). In summary, single-nuclei suspensions were combined with a master mix, loaded into microfluidic chips, and partitioned into gel beads-in-emulsion (GEMs). RNAs were captured and cDNAs made with primers containing a poly(dT) sequence, unique molecular identifier (UMI), GEM-specific barcode, and Illumina TruSeq adapter. After cDNA synthesis, GEMs were broken, cDNAs were amplified with 13 PCR cycles, and libraries were prepared from the amplified cDNA. After library construction, an additional 7-cycle PCR step was performed to replace Illumina sequencing adapters, and libraries were sequenced on the Singular Genomics G4 platform targeting at least 10,000 paired-end reads per nucleus. Data Processing and Quality Control - raw base call files were processed using Cell Ranger (v9.0.1) against the mm10 reference genome to generate gene-cell count matrices. Data Processing and Quality Control. Downstream analyses were performed in R (v4.4.3) using Seurat (v5.3.0) ^8^. Ambient RNA contamination was corrected using SoupX^9^ prior to filtering. Doublets were identified and removed using scDblFinder^10^. Cells were excluded based on the following criteria: <395 detected genes; >4716 detected genes; >15% mitochondrial transcripts. After filtering, 35,041 high-quality cells were retained for downstream analysis. Normalization and variance stabilization were performed using SCTransform with regression of mitochondrial percentage and sequencing depth. Samples were integrated using Harmony^11^ to correct for batch effects across biological replicates. Principal component analysis (PCA) was performed on the integrated object, and significant components were selected based on elbow plot inspection and variance explained. UMAP was used for visualization. Graph-based clustering was performed using Seurat’s FindNeighbors and FindClusters functions (resolution = 0.6). Cell types were annotated based on established canonical marker genes and cluster-specific differentially expressed genes. To preserve biological replication and avoid pseudoreplication, differential expression testing was performed using a pseudobulk approach implemented in muscat^12^. For each annotated cell type, raw counts were aggregated per biological replicate. Statistical testing was performed treating each animal as the unit of replication using edgeR/DESeq2-based modeling, with diet as the primary covariate. Multiple testing correction was performed using the Benjamini-Hochberg method. Genes with FDR-adjusted P < 0.05 were considered statistically significant. Differential abundance of cell populations was assessed by comparing the relative frequency of each annotated cell type per biological replicate between diets using Speckle ^13^. P values < 0.05 were considered significant. Raw and processed sequencing data have been deposited in GEO under accession number (will be provided upon acceptances). No regression of biological covariates (e.g., diet) was performed during integration to avoid removal of true biological signal.

**Osteoblast Experiment.** Three independent human osteoblast cell lines (PromoCell, Catalog no. C-12720, **Extended Data Table 5**) were selected, all harvested from the femoral head of Caucasian non-smokers with osteoarthrosis but not osteoporosis: male age 74 (lot no. 519Z062), female age 72 (lot no. 501Z014.2), and female age 64 (lot no. 498Z010.1). Upon confluency in Osteoblast Growth Medium, each cell line was treated with either 10 ng/mL of 5-HT (BioTechne, Catalog no. 3547/50), 50 ng/mL of 5-HT, 1 µg/mL of 1 µm polystyrene (PS) microspheres (Magsphere, Catalog no. PS001UM), 1 µg/mL of 5 µm PS microspheres (Magsphere, Catalog no. PS005UM), or vehicle control for 10 days in Osteoblast Growth Medium. Following this initial exposure, cells were maintained under the same treatment conditions in Osteoblast Mineralization Medium for an additional 21 days. At the conclusion of the treatment period, mineral deposition was assessed by staining cells with Alizarin Red S (pH 4.1) for 30 minutes at room temperature, protected from light. Cells were subsequently washed three times with Dulbecco’s phosphate-buffered saline (PBS) without Ca²⁺ or Mg²⁺, then counterstained with DAPI. Fluorescence intensity was quantified using a Cytation™ 5 plate reader at a resolution of 15x15 reads per well (alizarin 𝜆_𝑒𝑥_ = 496 nm and 𝜆_𝑒m_ = 616 nm; DAPI 𝜆_𝑒𝑥_ = 359 nm and 𝜆_𝑒m_ = 451 nm) to determine osteoblast mineralization relative to nuclei count.

**Statistical Analysis.** Statistical analysis was performed as described in figure legends and graphs generated display mean (±SD or SEM) and were obtained using GraphPad Prism software. If female and male data were analyzed separately, open circles, squares and triangles represent female mice whereas closed circles, squares and triangles represent male mice. The data were analyzed using two-tailed unpaired Student’s t test, a two-way ANOVA with Tukey’s multiple comparison test or described in the figure legend (GraphPad Prism v10.6.1 (892)).

**EXTENDED DATA**

EXTENDED DATA FIGURE 1


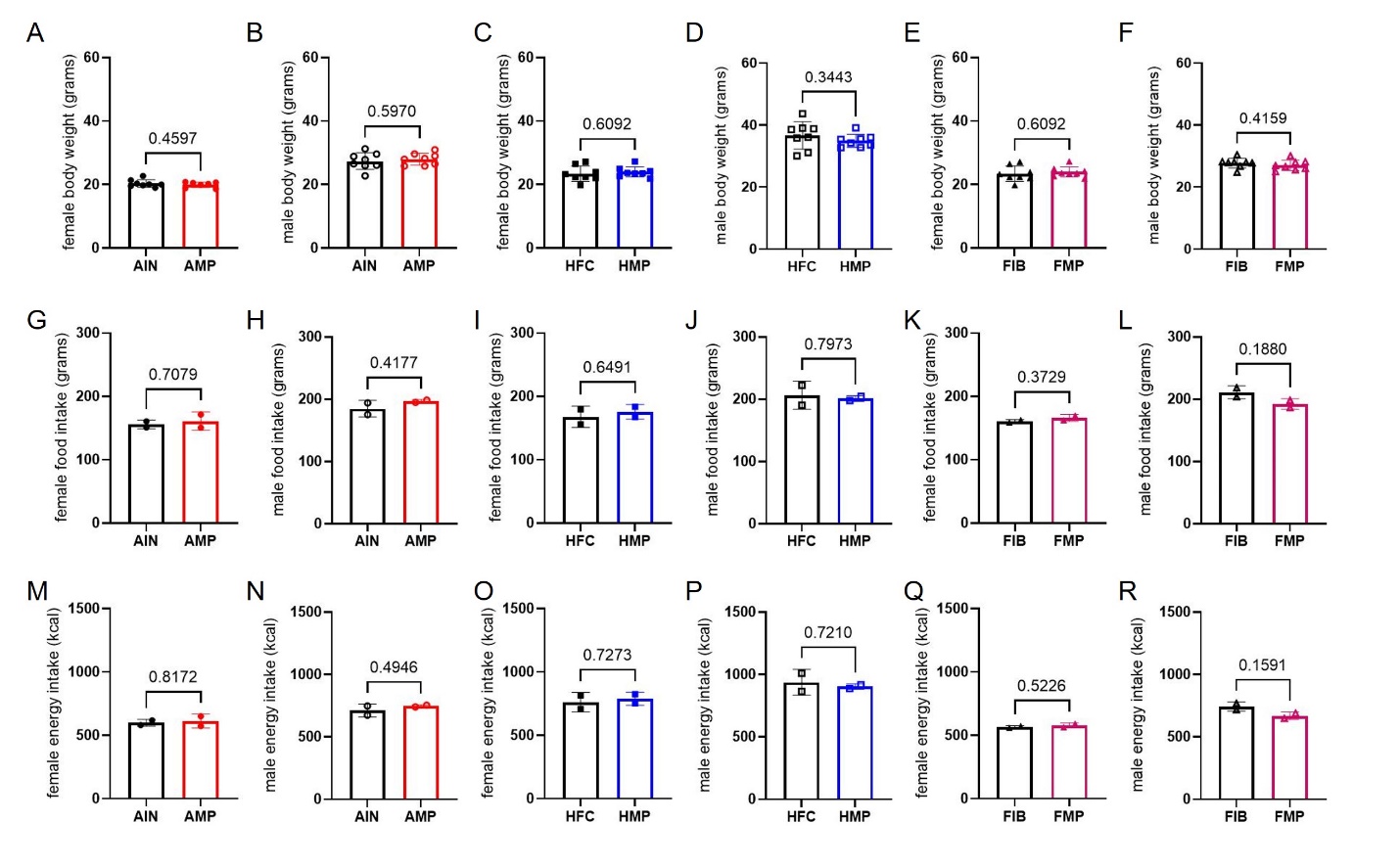


**Extended Data Figure 1.** Microplastic intake regardless of diet and did not affect body mass in female or male C57BL/6J mice. **a-f**, final body weights for basal (a, b), high-fat (c, d), and high-fiber (e, f) diets. **g-l**, total food intake for basal (g, h), high-fat (i, j), and high-fiber (k, l) diets. **m-r**, total energy intake for basal (m, n), high-fat (o, p), and high-fiber (q, r) diets. Open circles, squares and triangles represent female mice whereas closed circles, squares and triangles represent male mice. Data are mean ± SD; dots represent individual mice (n=8) or cage (n=0) and analyzed using two-tailed unpaired Student’s t test or Welch’s t test.

EXTENDED DATA FIGURE 2


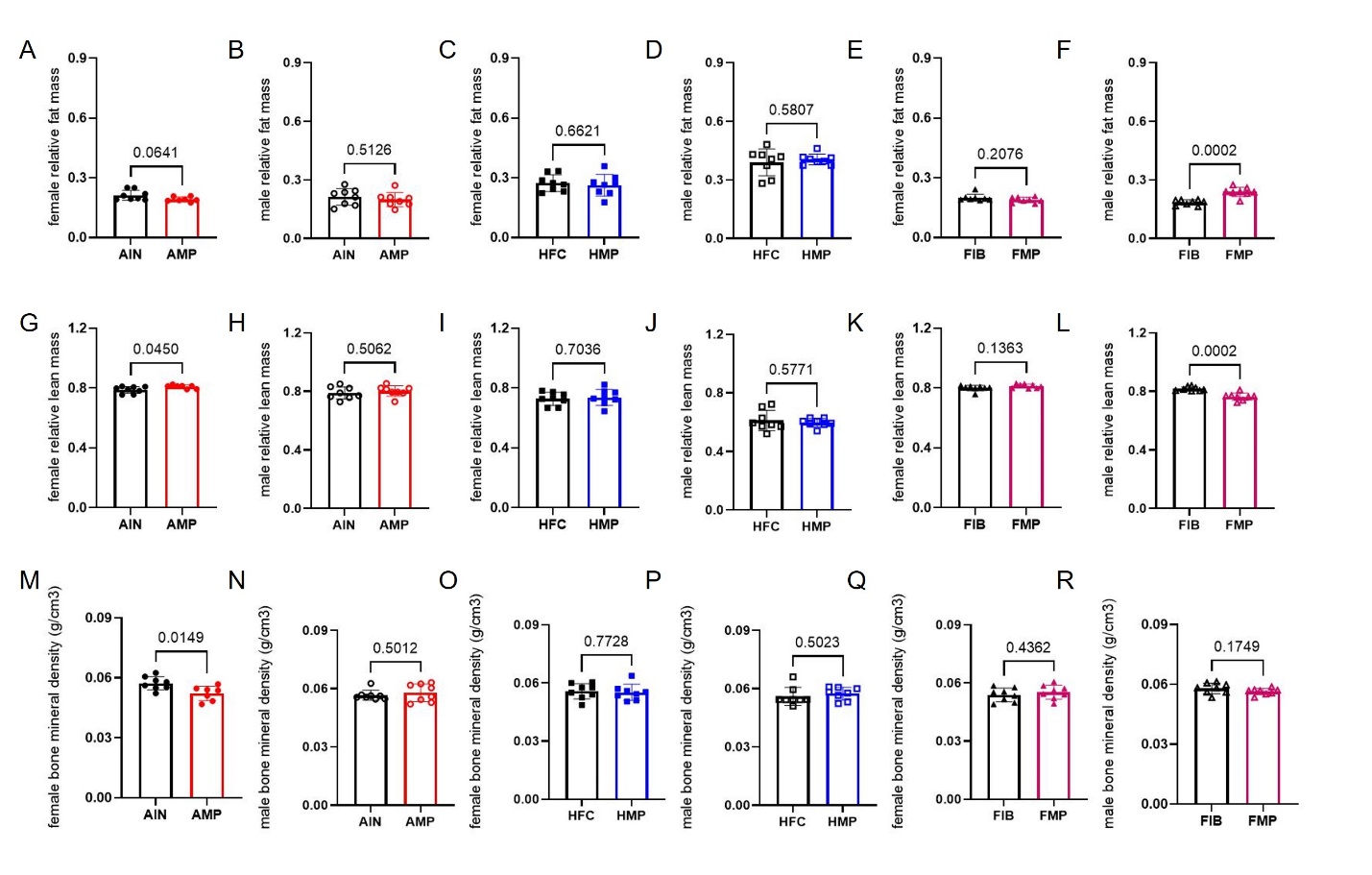


**Extended Data Figure 2.** Sex- and diet-dependent alterations following microplastic exposure in body composition and bone mineral density assessed by DEXA at 12 weeks. **a-f**, terminal fat mass for basal (a, b), high-fat (c, d), and high-fiber (e, f) diets. **g-l**, terminal lean mass for basal (g, h), high-fat (i, j), and high-fiber (k, l) diets. **m-r**, terminal bone mineral density for basal (m, n), high-fat (o, p), and high-fiber (q, r) diets. Open circles, squares and triangles represent female mice whereas closed circles, squares and triangles represent male mice. Data are mean ± SD; dots represent individual mice and analyzed using two-tailed unpaired Student’s t test or a Welch’s t test.

EXTENDED DATA FIGURE 3


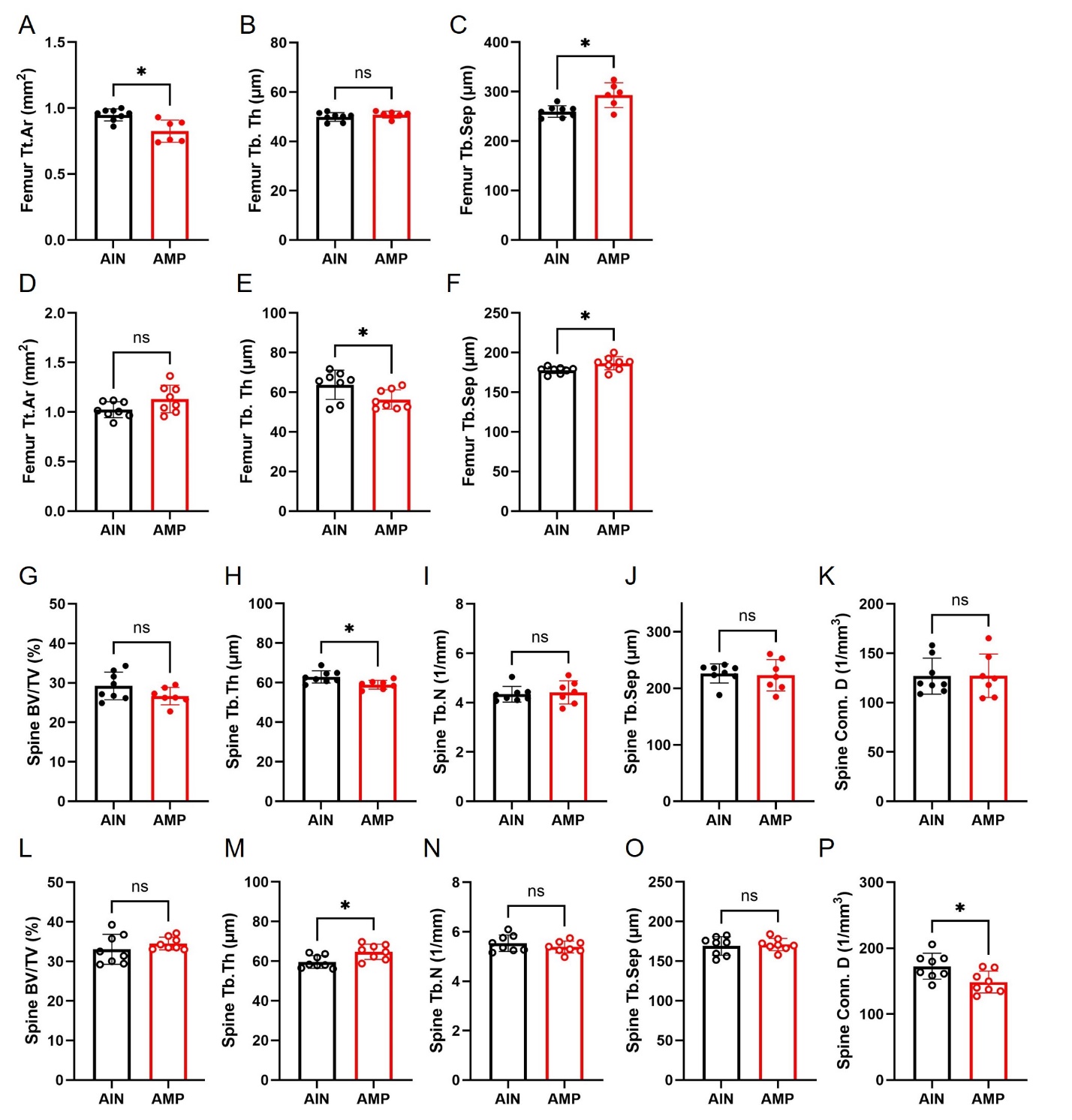


**Extended Data Figure 3. Dietary microplastic exposure disrupts vertebral bone microarchitecture in a sex-specific manner.** **a-c,** Femoral µCT analysis in females (closed circles) showing reduced (a) total area (Tt.Ar), (b) trabecular thickness (Tb.Th), (**c**) with increased trabecular separation (Tb.Sep). **d-f**, Femoral µCT analysis in males (open circles) showing (a) total area (Tt.Ar), (b) decreased trabecular thickness (Tb.Th), (**c**) with increased trabecular separation (Tb.Sep) in AMP-fed mice relative to AIN controls. **g-k,** Spinal µCT analysis in females (closed circles) demonstrating preserved vertebral parameters (**g, i-k**) but reduced spinal trabecular thickness (Tb.Th) (**h**), in AMP-fed mice. **l-o,** Spinal µCT analysis in males (open circles) demonstrating preserved vertebral parameters (**l, n, o**) but increased spinal trabecular thickness (Tb.Th) (m) and reduced spinal connective density (Conn.D) (p), in AMP-fed mice. Open circles represent female mice whereas closed circles represent male mice. Data are mean ± SD; dots represent individual mice. Statistical significance is indicated as *P < 0.05; ns, not significant.

EXTENDED DATA FIGURE 4


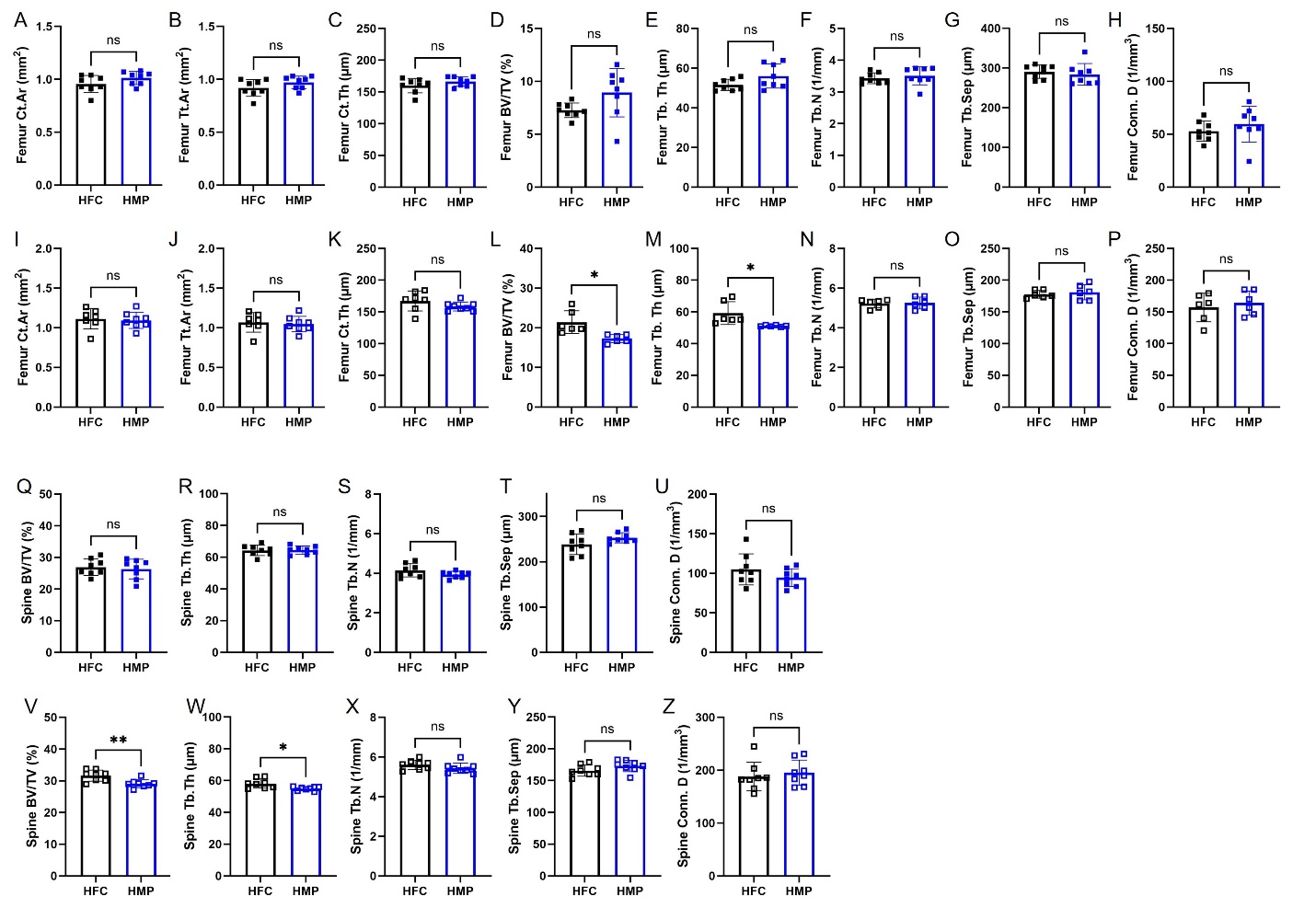


**Extended Data Figure 4. Dietary microplastic exposure disrupts bone microarchitecture in male mice on a high fat diet.** **a-h,** Femoral µCT analysis in females (closed squares) showing preserved vertebral parameters (Ct.Ar; Tt.Ar; Ct.Th;BV/TV; Tb.Th; Tb.N; Tb.Sep; and Conn.D) in HMP-fed mice relative to HFC controls. **i-p,** Femoral µCT analysis in males (open squares) demonstrating preserved vertebral parameters (Ct.Ar; Tt.Ar; Ct.Th;BV/TV; Tb.Th; Tb.N; Tb.Sep; and Conn.D), (**i-p**) but reduced femur bone volume (BV/TV) (**l)**, and trabecular thickness (Tb.Th) (**m**) in HMP-fed mice. **q-u,** Spinal µCT analysis in females (closed squares) demonstrating preserved vertebral parameters (BV/TV; Tb.Th; Tb.N; Tb.Sep; and Conn.D) in HMP-fed mice compared to HFC-fed mice. **v-z,** Spinal µCT analysis in males (open squares) demonstrating preserved vertebral parameters (BV/TV; Tb.Th; Tb.N; Tb.Sep; and Conn.D), (**x-z**) but decreased spinal bone volume (BV/TV) (v) and trabecular thickness (Tb.Th) (w), in HMP-fed mice. Open squares represent female mice whereas closed squares represent male mice. Data are mean ± SD; dots represent individual mice. Statistical significance is indicated as *P < 0.05, **P < 0.01; ns, not significant.

EXTENDED DATA FIGURE 5


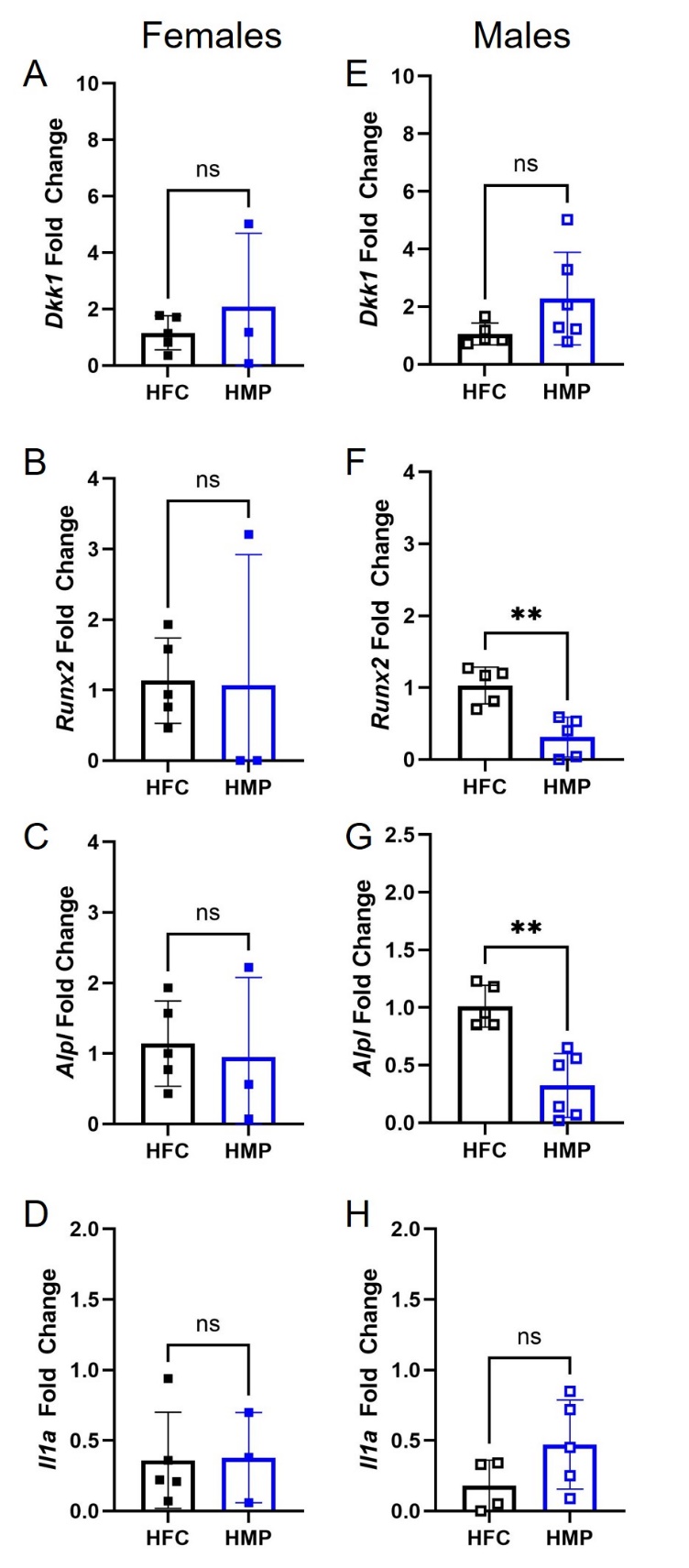


**Extended Data Figure 5. Dietary microplastic exposure alters transcriptional response in the bone of male mice on a high fat diet.**  **a-d**, Relative mRNA expression of Dkk1 (**a**), Runx2 (**b**), Alpl (**c**), and Il1a (**d**) in femoral bone from females. **e-h,** Relative mRNA expression of Dkk1 (**e**), Runx2 (**f**), Alpl (**g**), and Il1a (**h**) in femoral bone from males. Open squares represent female mice whereas closed squares represent male mice. Data are mean ± SD.; dots represent individual mice. Statistical significance is indicated as **P < 0.01; ns, not significant.

EXTENDED DATA FIGURE 6


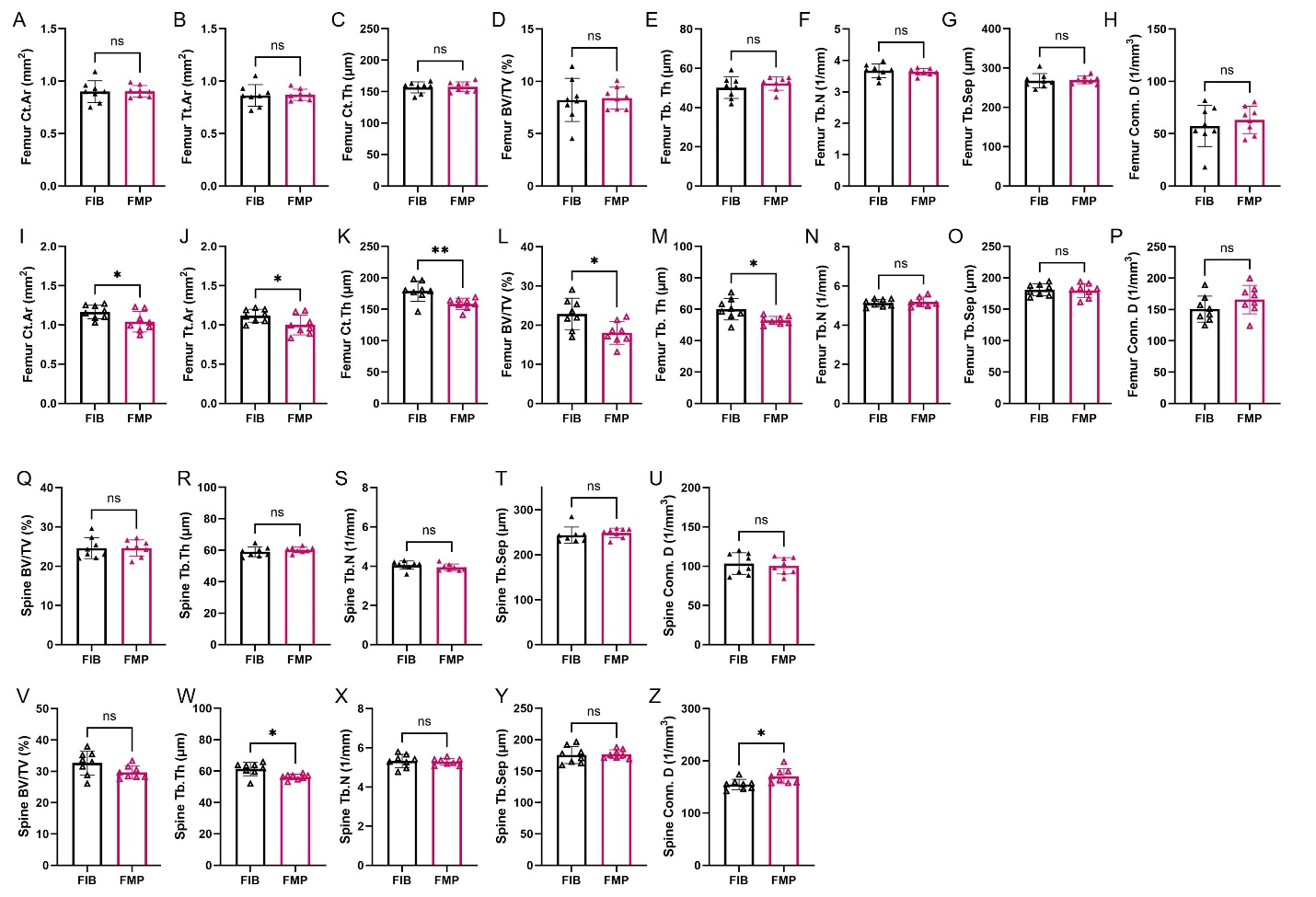


**Extended Data Figure 6. Dietary microplastic exposure disrupts bone microarchitecture in male mice on a high fiber diet.** **a-h,** Femoral µCT analysis in females (closed triangles) showing preserved vertebral parameters (Ct.Ar; Tt.Ar; Ct.Th;BV/TV; Tb.Th; Tb.N; Tb.Sep; and Conn.D) in FMP-fed mice relative to FIB controls. **i-p,** Femoral µCT analysis in males (open triangles) demonstrating preserved vertebral parameters (Ct.Ar; Tt.Ar; Ct.Th;BV/TV; Tb.Th; Tb.N; Tb.Sep; and Conn.D), (**n-p**), but reduced femur cortical area (Ct.Ar) (**l)**, total area (Tt.Ar) (**j**), cortical thickness (Ct.Th) (k), bone volume (BV/TV) (l), and trabecular thickness (Tb.Th) (m) in FMP-fed mice. **q-u,** Spinal µCT analysis in females (closed triangles) demonstrating preserved vertebral parameters (BV/TV; Tb.Th; Tb.N; Tb.Sep; and Conn.D) in FMP-fed mice compared to FIB-fed mice. **v-z,** Spinal µCT analysis in males (open triangles) demonstrating preserved vertebral parameters (BV/TV; Tb.Th; Tb.N; Tb.Sep; and Conn.D), (**v, x-y**) but decreased spinal trabecular thickness (Tb.Th) (w) and bone connective density (Conn.D) (z), in FMP-fed mice. Open triangles represent male mice whereas closed triangles represent female mice. Data are mean ± SD; dots represent individual mice. Statistical significance is indicated as *P < 0.05, **P < 0.01; ns, not significant.

EXTENDED DATA FIGURE 7


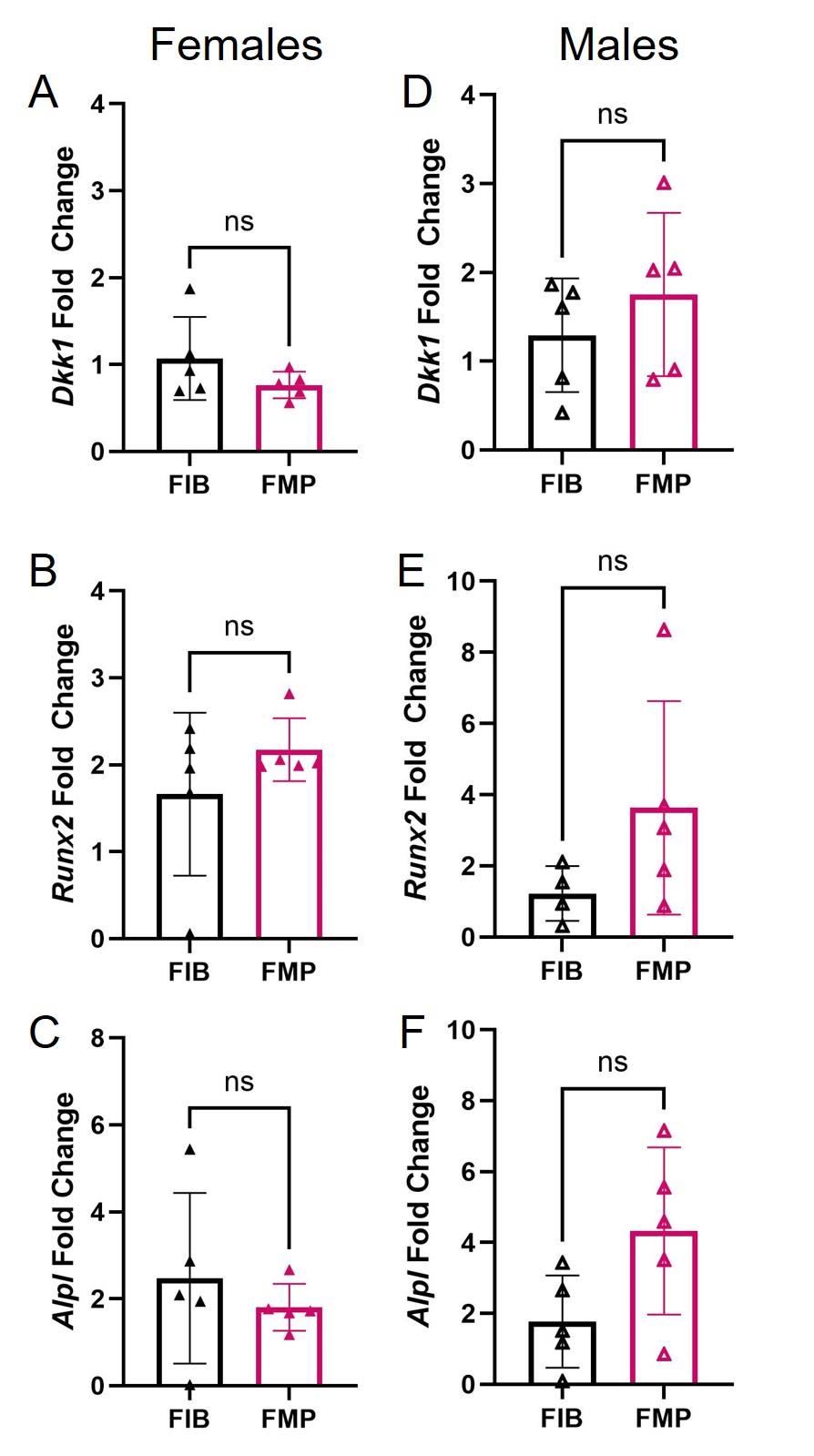


**Extended Data Figure 7. Dietary microplastic exposure do not alter transcriptional response in the bone of mice on a high fiber diet.**  **a-c**, Relative mRNA expression of Dkk1 (**a**), Runx2 (**b**), Alpl (**c**) in femoral bone from females. **d-f,** Relative mRNA expression of Dkk1 (**d**), Runx2 (**e**), Alpl (**f**), and in femoral bone from males. Open triangles represent male mice whereas closed triangles represent female mice. Data are mean ± SD; dots represent individual mice. Statistical significance is indicated as ns, not significant.

EXTENDED DATA FIGURE 8


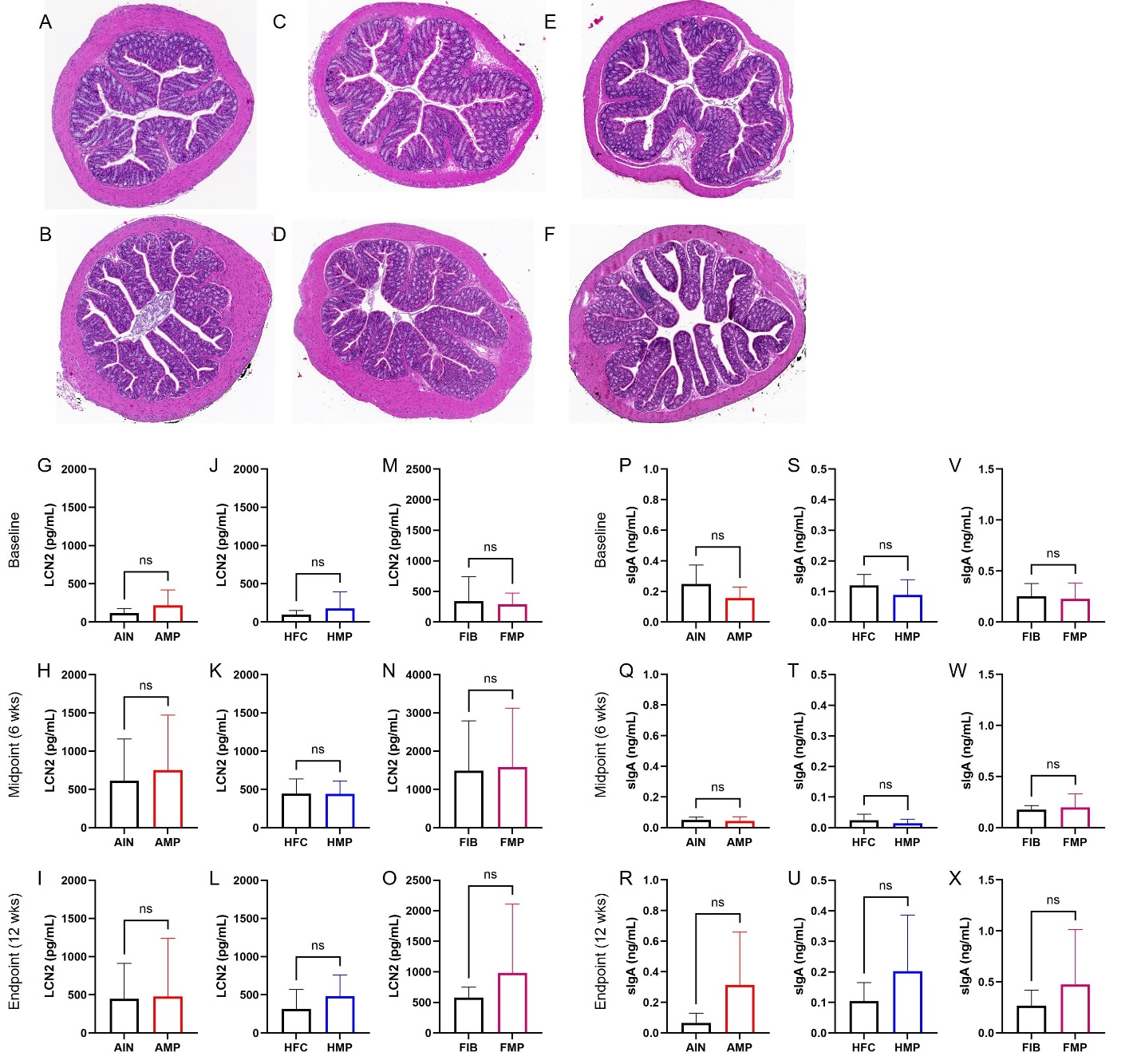


**Extended Data Figure 8. Dietary microplastic exposure does not induce overt intestinal inflammation. a-f,** Representative H&E-stained colon sections from mice on a control diet, AIN (a), control diet with MPs, AMP (b), high fat/high cholesterol diet, HFC (c), high fat/high cholesterol diet with MPs, HMP (d), high fiber diet, FIB (e), and high fiber diet with MPs, FMP (f). **g-o**, LCN-2 protein (pg/mL) measured across weeks -0 (g, j, m), -6 (h, k, n) and -12 (i, l, o) to evaluate intestinal inflammation in the stool of mice from each of the diets. ). **p-x**, sIgA antibody (ng/mL) measured across weeks -0 (p, s, v), -6 (q, t, v) and -12 (r, u, x) to evaluate intestinal inflammation in the stool of mice. Data are mean ± SD (n=8), statistical significance is indicated as ns, not significant.

EXTENDED DATA FIGURE 9


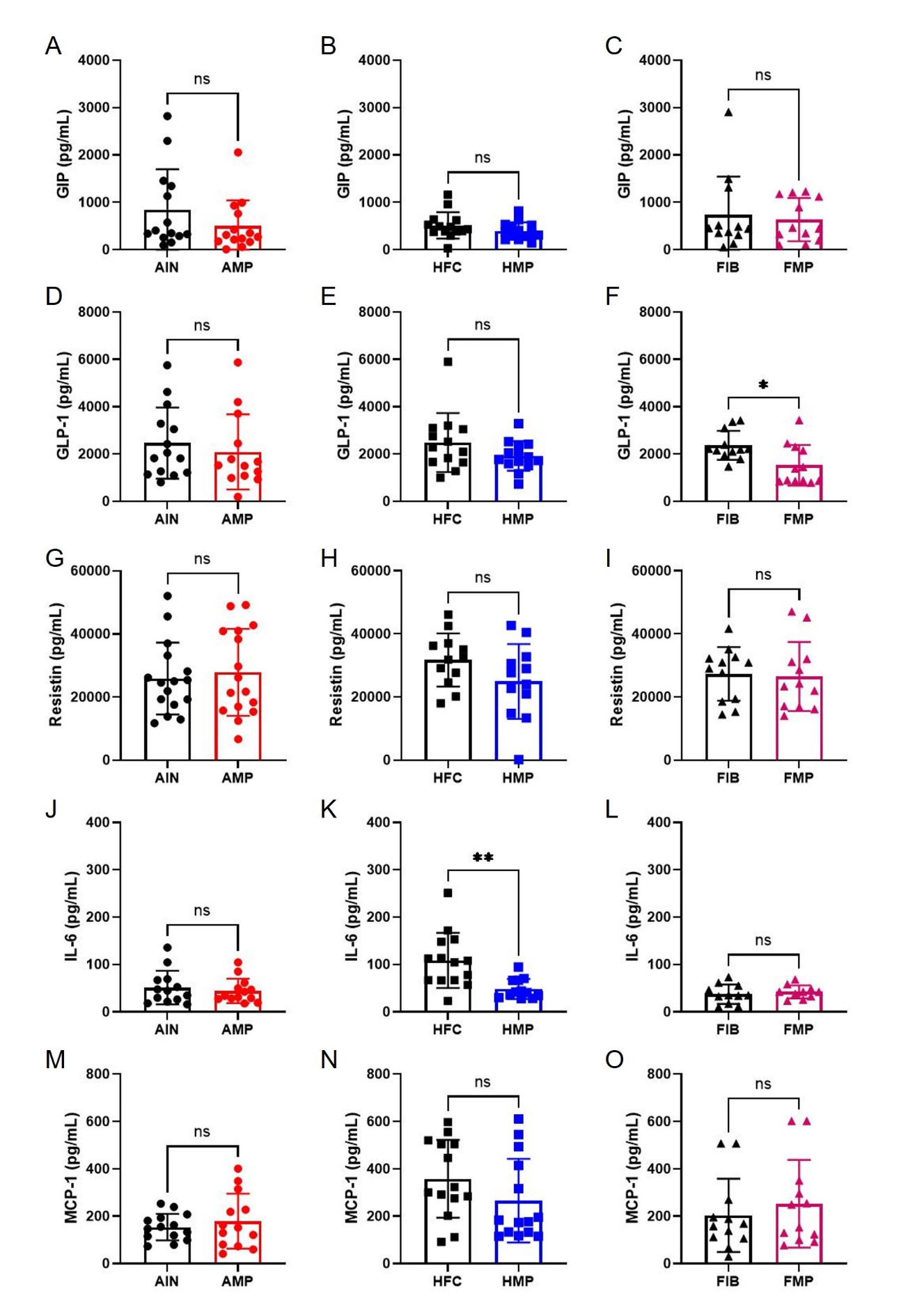


**Extended Data Figure 9. Dietary microplastic exposure does not cause broad hormonal changes in the plasma of mice. a-o,** Circulating cytokines and hormones were assessed in plasma from mice on a control diet (AIN), control diet with MPs (AMP), high fat/high cholesterol diet (HFC), high fat/high cholesterol diet with MPs (HMP), high fiber diet (FIB), and high fiber diet with MPs (FMP). (a-c) GIP; (e-f) GLP-1; (g-i) Resistan; (j-l) IL-6; (m-o) MCP-1. Data are mean ± SD; dots represent individual mice. Data analyzed using Student’s t-test. Statistical significance is indicated as **P < 0.01; ns, not significant.

EXTENDED DATA FIGURE 10


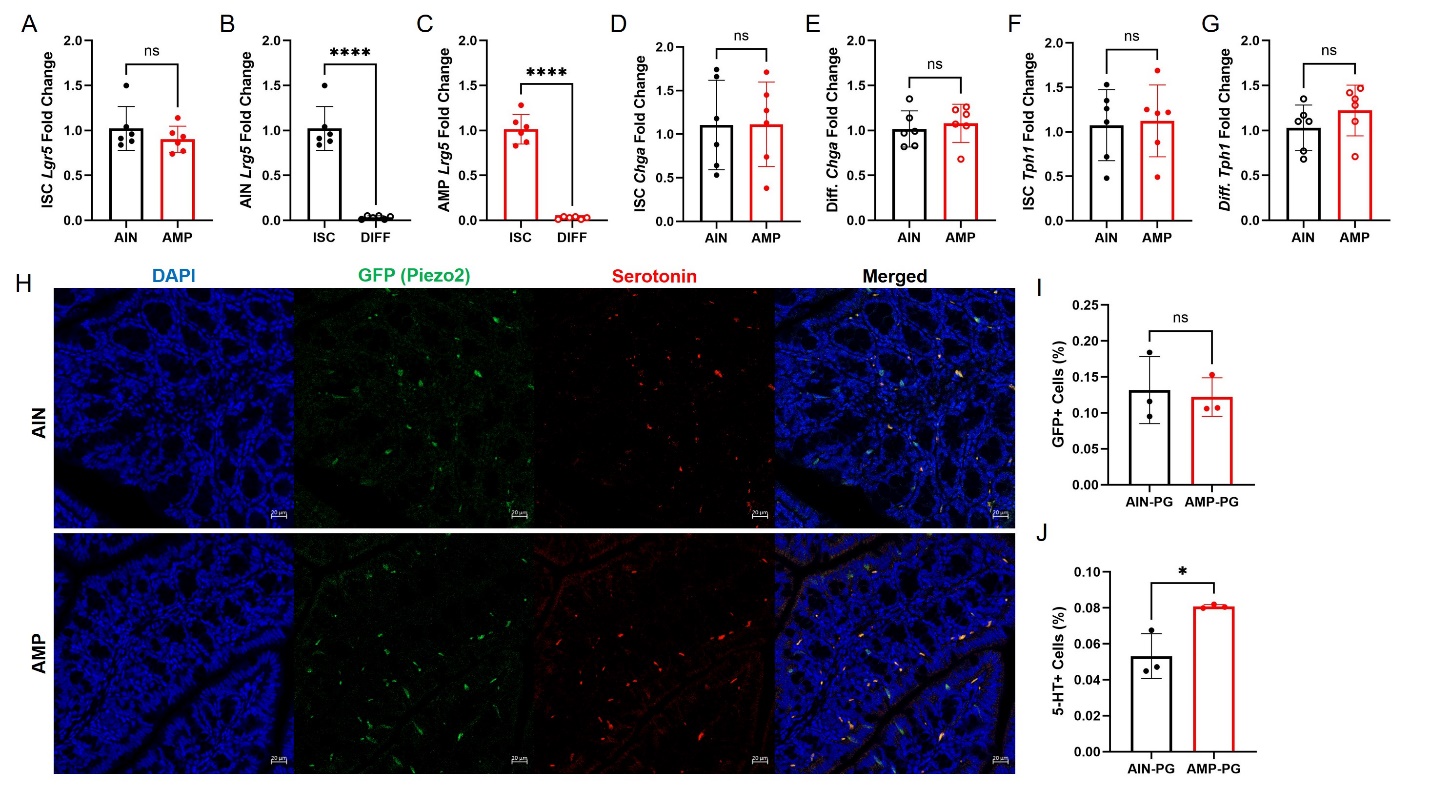


**Extended Data Figure 10. Organoid and Piezo2-GFP mice examination. a-g, Stem and enteroendocrine cell transcripts from colonic organoids derived from mice on a control diet without (AIN) or with MPs (AMP). a, *Lgr5*, an intestinal stem cell marker, expression in colonic organoids in a stem state comparing AIN- and AMP-derived colonic organoids. b-c, *Lgr5* expression comparing colonic organoids in a stem state to differentiated (DIFF) colonic organoids in (b) AIN and (c) AMP samples. d-e, *Chga* (chromogranin A), a pan enteroendocrine cell marker assessed** at (d) stem state in AIN- and AMP-**derived colonic organoids or (e) and** in differentiated colonic organoids derived from AIN- and AMP-fed mice. **f-g, (f) *Tph1* (**Tryptophan Hydroxylase 1), expressed in enterochromaffin cells, **assessed** at (f) stem state in AIN- and AMP-**derived colonic organoids or (g) and** in differentiated colonic organoids derived from AIN- and AMP-fed mice. **h,** Representative immunofluorescence images of proximal colon from AIN- and AMP-fed Piezo2-GFP reporter mice, stained for nuclei (DAPI, blue), GFP (green), and serotonin (5-HT, red). Merged images are shown. Scale bars, 20 μm. **i,j,** Quantification of GFP^+^ enterochromaffin cell abundance (i) and serotonin immunoreactivity per GFP^+^ cell (j) in colonic epithelium from AIN- and AMP-fed mice. Data are shown as mean ± s.d.; dots represent individual mice or independent organoid cultures, as indicated. Statistical significance is indicated as *P < 0.05, ****P < 0.001; ns, not significant.

EXTENDED DATA FIGURE 11


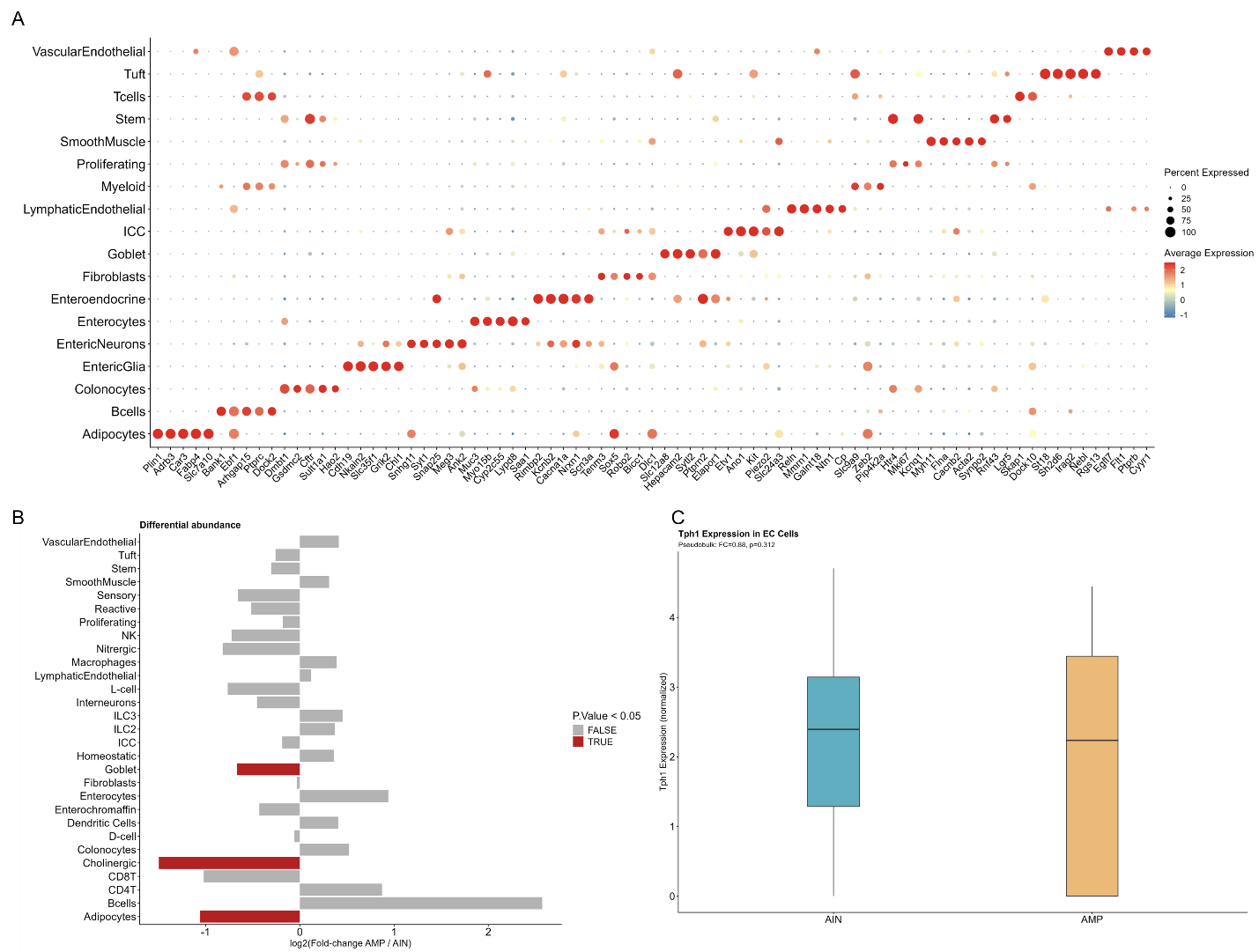


**Extended Data Figure 11. Single nuclei RNA sequencing and cell specific transcriptional expression. a, Dot plot depicting mean expression and percent-expressing cells across all different cells types found within the colon indicating the highest expressed markers per cell type. b, Differential abundance through log2(fold change) where goblet, cholinergic, and adipocytes were more concentrated in microplastic treated colons (AMP) as compared to the control (AIN). c, Normalized *Tph1* expression on enterochromaffin cells in AIN and AMP colons.**

EXTENDED DATA FIGURE 12


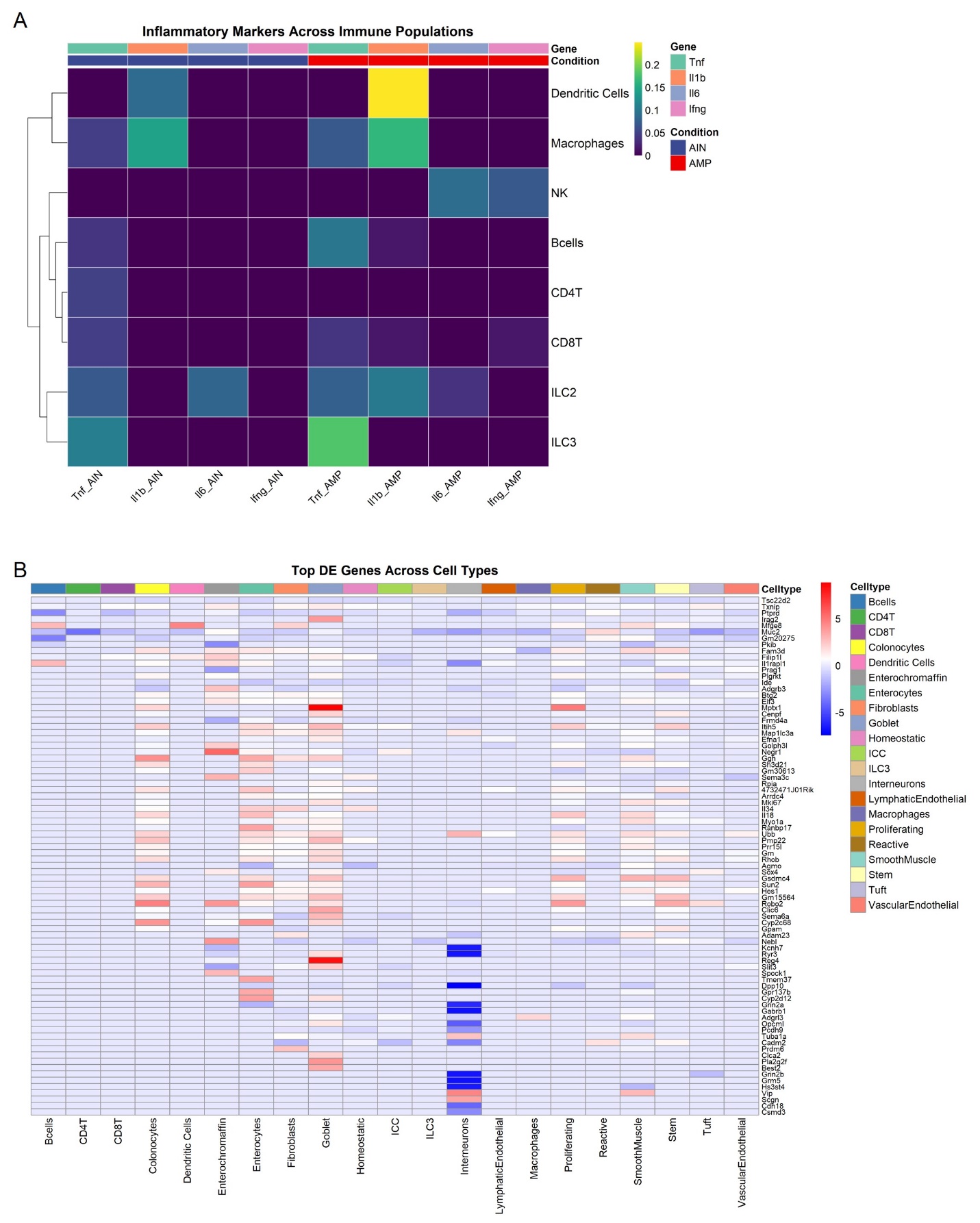


**Extended Data Figure 12. Single nuceli RNA sequencing and inflammatory cell specific transcriptional expression. a, heat map evaluating inflammatory markers across immune populations. b, heat map displaying the top differentially expressed genes across all cell types.**

EXTENDED DATA FIGURE 13


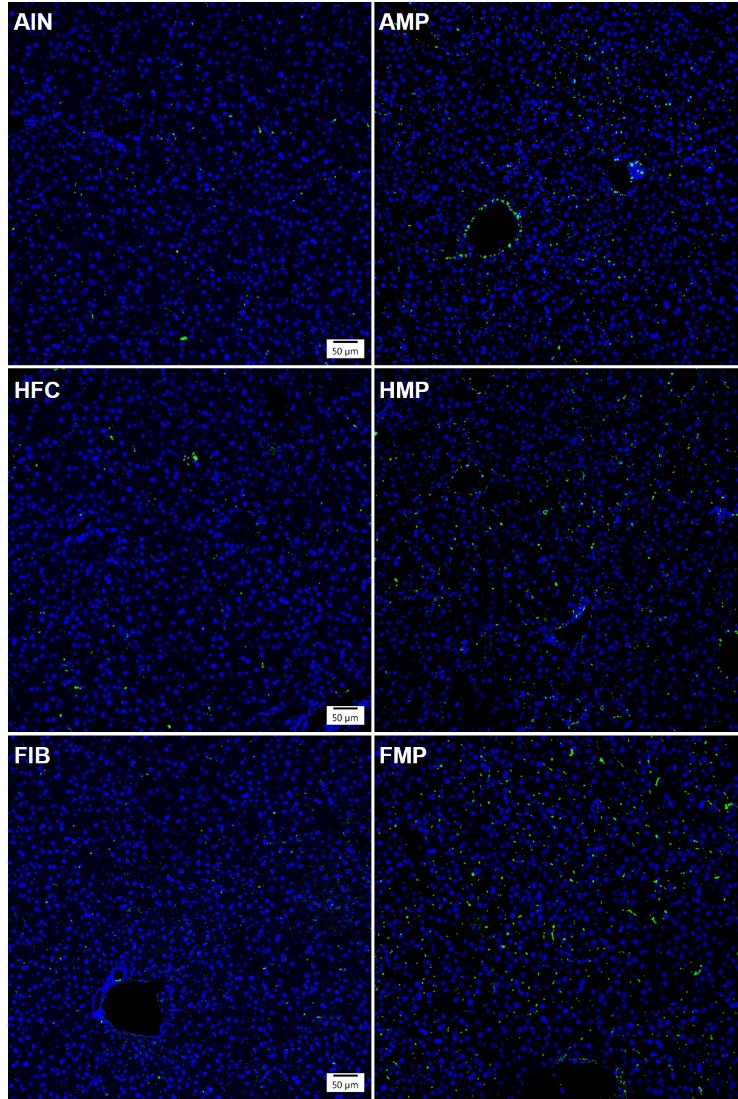


**Extended Data Figure 13. Microplastic (MP) detection in liver tissues from MP-diet exposure.** Representative fluorescence images of histological sections from livers from our various groups (AIN, AMP, HFC, HMP, FIB, and FMP) stained with DAPI (blue) to label nuclei and a conjugated polymer nanoparticle-based MP dye (MP-DYE, green). Objective magnification: 20X. Scale bars, 50 µm.

EXTENDED DATA FIGURE 14


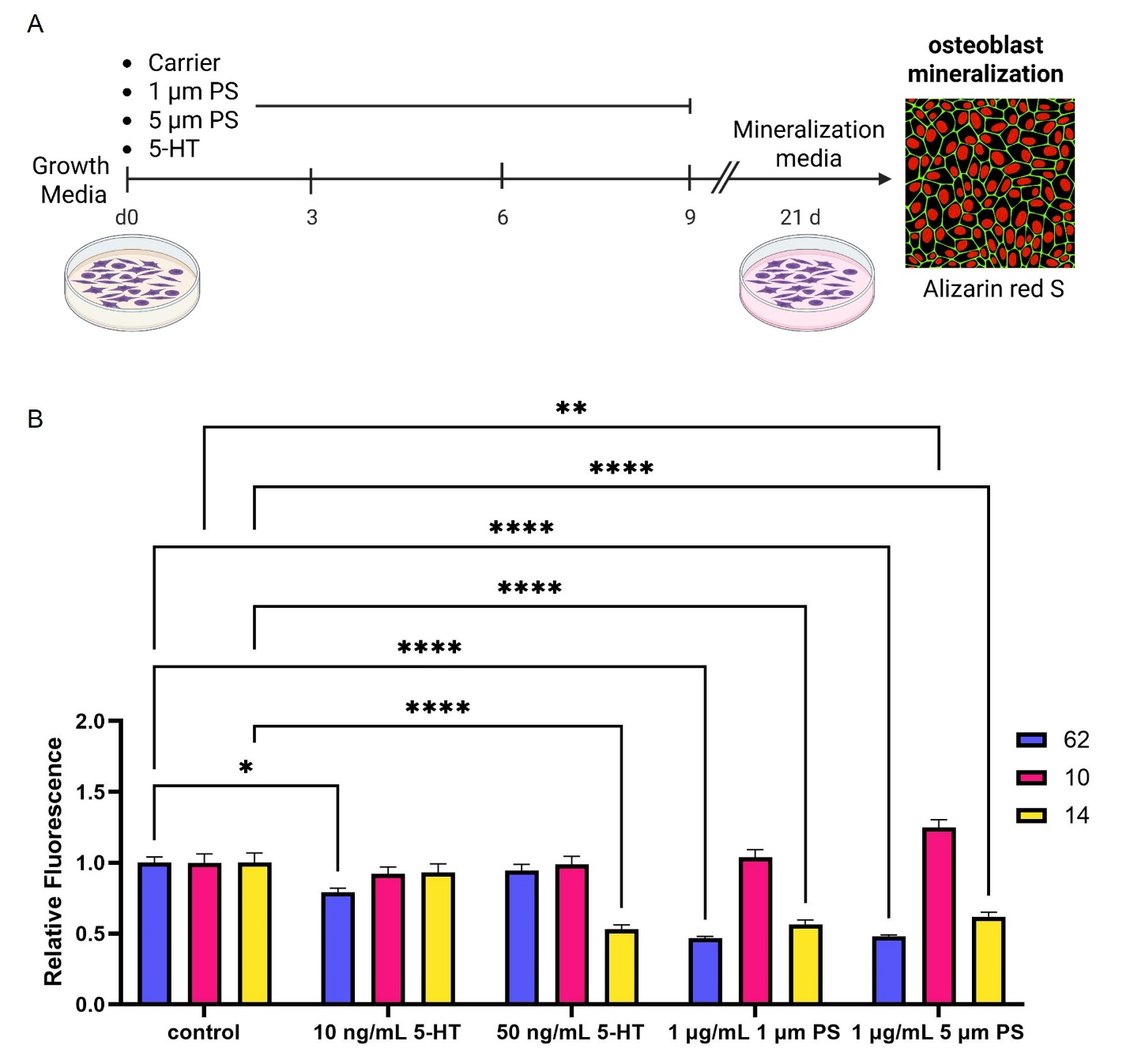


**Extended Data Figure 14. I**nter-individual variability in osteoblast mineralization following polystyrene microsphere and serotonin exposure. **a, *in vitro* approach where t**hree human osteoblast cell lines (identifiers 62, 10, and 14 are individual donors, donor information can be found in **Extended Data Table 5**) were treated with either 10 ng/mL of 5-HT, 50 ng/mL of 5-HT, 1 µg/mL of 1 µm PS, 1 µg/mL of 5 µm PS, or vehicle control for 10 days (day 0-9) in Osteoblast Growth Medium followed by 21 days is Osteoblast Mineralization Medium, then stained with Alizarin Red S and DAPI. **b**, Alizarin:DAPI fluorescence ratios for each treatment group were normalized to DMSO controls; data are shown as mean ± SEM and analyzed using a two-way ANOVA with Tukey’s multiple comparison test.

REFERENCES

1. Senathirajah, K. *et al.* Estimation of the mass of microplastics ingested - A pivotal first step towards human health risk assessment. *J Hazard Mater* **404**, 124004 (2021).

2. Merkley, S.D. *et al.* Non-autophagy Role of Atg5 and NBR1 in Unconventional Secretion of IL-12 Prevents Gut Dysbiosis and Inflammation. *J Crohns Colitis* **16**, 259-274 (2022).

3. Erben, U. *et al.* A guide to histomorphological evaluation of intestinal inflammation in mouse models. *Int J Clin Exp Pathol* **7**, 4557-4576 (2014).

4. In, J.G. *et al.* Epithelial WNT2B and Desert Hedgehog Are Necessary for Human Colonoid Regeneration after Bacterial Cytotoxin Injury. *iScience* **23**, 101618 (2020).

5. Atanga, R. *et al.* Inflammatory macrophages prevent colonic goblet and enteroendocrine cell differentiation through Notch signaling. *bioRxiv* (2023).

6. Atanga, R., Parra, A.S. & In, J.G. Efficient RNA and RNA-protein co-detection in 3D colonoids by whole-mount staining. *STAR Protoc* **3**, 101775 (2022).

7. Atanga, R. *et al.* Single Cell Analysis of Human Colonoids Exposed to Uranium-Bearing Dust. *Environ Health Perspect* **132**, 57006 (2024).

8. Hao, Y. *et al.* Dictionary learning for integrative, multimodal and scalable single-cell analysis. *Nat Biotechnol* **42**, 293-304 (2024).

9. Young, M.D. & Behjati, S. SoupX removes ambient RNA contamination from droplet-based single-cell RNA sequencing data. *Gigascience* **9** (2020).

10. Germain, P.L., Lun, A., Garcia Meixide, C., Macnair, W. & Robinson, M.D. Doublet identification in single-cell sequencing data using scDblFinder. *F1000Res* **10**, 979 (2021).

11. Korsunsky, I. *et al.* Fast, sensitive and accurate integration of single-cell data with Harmony. *Nat Methods* **16**, 1289-1296 (2019).

12. Crowell, H.L. *et al.* muscat detects subpopulation-specific state transitions from multi-sample multi-condition single-cell transcriptomics data. *Nat Commun* **11**, 6077 (2020).

13. Phipson, B. *et al.* propeller: testing for differences in cell type proportions in single cell data. *Bioinformatics* **38**, 4720-4726 (2022).
